# Metabolic Toxification of 1,2-Unsaturated Pyrrolizidine Alkaloids Causes Human Hepatic Sinusoidal Obstruction Syndrome: The Update

**DOI:** 10.3390/ijms221910419

**Published:** 2021-09-27

**Authors:** Rolf Teschke, Noudeng Vongdala, Nguyen Van Quan, Tran Ngoc Quy, Tran Dang Xuan

**Affiliations:** 1 Department of Internal Medicine II, Division of Gastroenterology and Hepatology, Klinikum Hanau, Teaching Hospital of the Medical Faculty, Goethe University Frankfurt/Main, 63450 Hanau, Germany; 2 Laboratory of Plant Physiology and Biochemistry, Graduate School of Advanced Science and Engineering, Hiroshima University, Hiroshima 739-8529, Japan; vongdala1986@gmail.com (N.V.); nvquan@hiroshima-u.ac.jp (N.V.Q.); tnquy@ctu.edu.vn (T.N.Q.); tdxuan@hiroshima-u.ac.jp (T.D.X.)

**Keywords:** 1,2-unsaturated pyrrolizidine alkaloids, hepatic sinusoidal obstruction syndrome, HSOS, Roussel Uclaf Causality Assessment Method, herb-induced liver injury, HILI

## Abstract

Saturated and unsaturated pyrrolizidine alkaloids (PAs) are present in more than 6000 plant species growing in countries all over the world. They have a typical heterocyclic structure in common, but differ in their potential toxicity, depending on the presence or absence of a double bond between C1 and C2. Fortunately, most plants contain saturated PAs without this double bond and are therefore not toxic for consumption by humans or animals. In a minority of plants, however, PAs with this double bond between C1 and C2 exhibit strong hepatotoxic, genotoxic, cytotoxic, neurotoxic, and tumorigenic potentials. If consumed in error and in large emouns, plants with 1,2-unsaturated PAs induce metabolic breaking-off of the double bonds of the unsaturated PAs, generating PA radicals that may trigger severe liver injury through a process involving microsomal P450 (CYP), with preference of its isoforms CYP 2A6, CYP 3A4, and CYP 3A5. This toxifying CYP-dependent conversion occurs primarily in the endoplasmic reticulum of the hepatocytes equivalent to the microsomal fraction. Toxified PAs injure the protein membranes of hepatocytes, and after passing their plasma membranes, more so the liver sinusoidal endothelial cells (LSECs), leading to life-threatening hepatic sinusoidal obstruction syndrome (HSOS). This injury is easily diagnosed by blood pyrrolizidine protein adducts, which are perfect diagnostic biomarkers, supporting causality evaluation using the updated RUCAM (Roussel Uclaf Causality Assessment Method). HSOS is clinically characterized by weight gain due to fluid accumulation (ascites, pleural effusion, and edema), and may lead to acute liver failure, liver transplantation, or death. In conclusion, plant-derived PAs with a double bond between C1 and C2 are potentially hepatotoxic after metabolic removal of the double bond, and may cause PA-HSOS with a potential lethal outcome, even if PA consumption is stopped.

## 1. Introduction

Patients with acute or chronic liver diseases represent a clinical challenge if toxins are suspected, as possible causes are diverse, such as plant-derived 1,2-unsaturated pyrrolizidine alkaloids (PAs) [1,2,3,4,5,6], various other phytochemicals [7,8,9,10,11], ethanol [12,13,14,15], conventional drugs [16,17,18,19], or industrial aliphatic halogenated hydrocarbons like carbon tetrachloride [20]. Most of these toxins share the common feature of metabolic activation as a prerequisite for their liver-injurious potential, but they may differ regarding their chemical structures, types of liver injury, and typical clinical features. Toxic liver injury must be differentiated regarding each causative toxin because clinical evaluation, including formal causality assessement and therapy, can vary substantially. For instance, following prolonged use of high amounts of alcohol, alcoholic liver disease can develop with disease stages ranging from alcoholic fatty liver [13,14,15] to alcoholic cirrhosis, including the risk of primary hepatocellular carcinoma (HCC) [15]. At recommended daily doses or overdosed, drugs may lead to drug-induced liver injury (DILI) with the risk of acute liver failure, requiring liver transplantation [16,17,18,19]. Finally, herbal products used as prescribed or over-the-counter medicines are causes of rare but potentially life-threatening herb-induced liver injury (HILI) [7,8,9,10,11]. Among the plants leading to most severe liver injury are those with 1,2-unsaturated PAs as ingredients. By chemotaxonomical calculation, PAs are estimated to be present in more than 6000 plant species, which is equal to around 3% of all flowering plants, a fact published for the first time in 1980, by Culvenor [1]. Their use may result in a specific liver disease called hepatic sinusoidal obstruction syndrome (HSOS) [3,4,5,6].

Much progress has been made within the last years regarding 1,2-unsaturated PAs metabolic conversion in liver cells via the microsomal cytochrome P450 (CYP) isoforms to toxic intermediates, injurious, mainly, to liver sinusoidal endothelial cells (LSECs) as the target cells of HSOS, differing thereby from other toxins attacking primarily liver parenchymal cells rather than the LSECs. In the past, HSOS was difficult to diagnose, an issue now solved by diagnostic advances in new biomarkers, evidence based diagnostic criteria, and the use of robust causality assessment methods such as the RUCAM (Roussel Uclaf Causality Assessment Method) as part of artificial intelligence (AI) principles.

In the present review article, we focus on recent developments on the molecular and metabolic events leading to PA-induced HSOS and its worldwide occurrence as sporadic cases or major disease clusters. Finally, the most important clinical features will be discussed.

## 2. Literature Search and Source

The PubMed database was searched for articles published preferentially during the last 5 years, in line with requirements of the editorial office by using the following key terms: herb-induced liver injury (HILI); hepatic sinusoidal obstruction syndrome (HSOS); pyrrolizidine alkaloids (PAs); Roussel Uclaf Causality Assessment Method (RUCAM). Only reports in English were selected and incorporated in this review article, containing the most relevant and recent publications, with the limitation that some important earlier publications could, regretfully, not be included.

## 3. Regulatory Approaches

Based on compelling evidence that 1,2-unsaturated PAs may cause toxicity, including liver injury dependent on the dose and duration of uptake, attempts to define the risk for humans exposed to PAs have been made in 2016 by the German Federal Institute for Risk Assessments (BfR in short for Bundesinstitut für Risikobewertung) [21], in 2020, by the regulatory European Medicines Agency (EMA) [22], again in 2020, by the European Food Safety Authority (EFSA) [23], and additionally in 2020 by the EU Commission [24]. Of course, many attempts at defining this risk have been undertaken before, but discussion on these issues is outside the frame of this article. The prevailing international sentiment is a disharmonious impression of the data’s variability and is now associated with permanent amendments, despite marginal improvements in data quality. Indeed, the scientific community is overrun by regulatory evaluations and conclusions, despite little evidence that food, consumed in Western countries and contaminated with PAs, does really cause HSOS in those consumers. Even worse, how can we be sure that unsaturated PAs cause malignancies such as HCC by tackling, specifically, liver parenchymal cells, or hemangiosarcoma by tackling, specifically, LSECs in patients living in the real world and consuming products containing various amounts of unsaturated PAs? There is no question: defining regulatory risks as precautionary measures is necessary, but should be accompanied by critical information.

In more detail, considering oral use and the BMDL_10_ (Bench Mark Dose Lower Confidence Limit), risk assessment by the EFSA deduced a BMDL_10_ of 237 μg/kg for all 1,2-unsaturated PAs, assuming equal potency [23]. However, there is much uncertainty about the extent by which toxicity can be affected by particular substitutions within the chemical structure of unsaturated PAs or by variations in biological activity among humans. According to the International Conferences on Harmonization (ICH), this BMDL_10_ must be divided by 10,000 to achieve the acceptable intake of 0.0237 μg/kg body weight, and assuming a 50 kg person, rather than 70 kg, as is the most commonly accepted weight; this would mean a daily intake of 1.0 μg per day for adults with this body weight [23]. The EMA provided additional details from Germany, published in 1992, by clarifying that for medicinal products containing PAs, the maximum daily dose of PAs for internal use is set at 1.0 μg for a duration of a maximum of 6 weeks per year, and 0.1 μg without any limitation on duration [22]. The presented value of 0.0237 µg/kg body weight is not a BMDL_10,_ as daily intake is not considered at this point, although the margin of exposure (MOE) approach is used for chronic consumption. Additionally, the presented value should not be considered as a regulatory limit. Indeed, considering an MOE of 10,000 and the actual BMDL_10_ of 237 μg/kg BW/d, an overall PA intake of more than 0.024 µg/kg BW/d can be seen as a threshold for beginning risk management. Additionally, a common procedure for assessing the acute risk (non-neoplastic damages) of PA intake is the health-based guidance value (HBGV) of 0.1 µg PA/kg body weight, using the NOAEL of 10 μg/kg BW/d and applying an uncertainty factor of 100 (see BfR, Federal Institute for Risk Assessment, Opinion No. 030/2016) [21]. Even more important, regulatory limits for PAs in (herbal) teas and spices were recently implemented in Commission Regulation (EC) No 1881/2006 (see Commission Regulation [EU] 2020/2040) [24].

## 4. Chemical Structure of a Typical 1,2-Unsaturated PA

**Figure 1:**
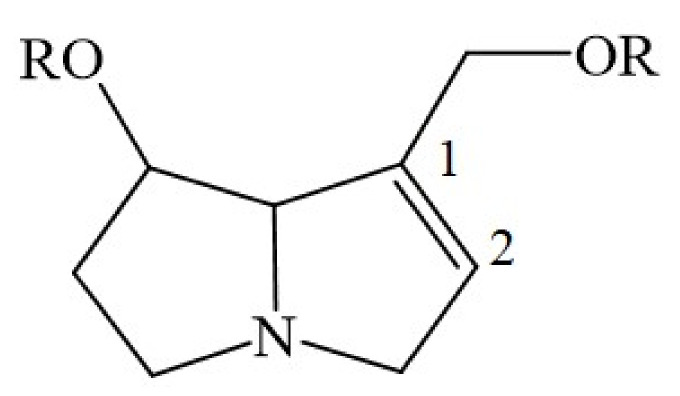
Prototypical basic structure of a toxic 1,2-unsaturated pyrrolizidine alkaloid; a sketeletal formula of retronecine, a PA found in the Common groundsel (*Senecio vulgaris*) and comfrey (*Symphytum* spp.). R illustrates the position of the different necic acids. Of note, the chemical structure of non-toxic PAs is similar to the structure shown above except that the double bond between C1 and C2 is missing. Structure was composed from various publications [1,2,3,4,5,6,21,22,23,24].

Synthesized by plants, all 1,2-unsaturated PAs share a basic chemical structure, as published in relevant reports [1,2,3,4,5,6,21,22,23,24]. The liver toxicity of unsaturated PAs is caused by the double bond between C1 and C2 (Figure 1).

## 5. Global Presence and Use of Plants Containing PAs

### 5.1. General Aspects

Plants containing PAs grow worldwide, both in tropical and non-tropical regions [3,4]. Their PAs are reported to act as a defense mechanism against insect herbivores by preventing plant damage and death [25]. As a consequence, this defense property allows for continued plant growth rather than reduced growth and is likely associated with the disadvantage of further propagation of PA-containing plants as weeds. Although only about 3% of flowering plants contain PAs [1], these potentially hepatotoxic plants have attracted much interest from scientists, epidemiologists, physicians, herbalists, regulators, and manufacturers [1,2,3,4,5,6,21,22,23,24]. In particular, listings of individual plants synthesizing PAs are carefully cited in several publications and available to interested readers. Such plants are known for their biodiversity, and PAs are characterized for their diversity of chemical structures, synthesizing pathways, and metabolic steps leading to toxicity [22,23,24].

### 5.2. Feed Products Containing PAs

Studies on samples in the Netherlands revealed variable amounts of PAs in feed products [23]. Some products showed no PAs at all, suggesting their lack of production or absent contamination with PA-containing plant parts; in other products, such as grass and hay, moderate levels are found, as opposed to the unexplained very high levels in Lucerne (alfalfa) (Table 1) [23].

The origin of PAs in feed has not thoroughly been investigated, but three possibilities are to be considered: first, plants synthesize PAs; second, plants take up PAs via horizontal transfer from the soil contaminated with PAs originating from nearby PA-containing plants; or third, plants become contaminated with PAs during processing plant products. Of interest for humans, feed containing PAs has variable influence on PA levels of animal products [22]. For instance, when PA-containing plants were given to laying hens, different PA levels were found in their eggs, ranging from not-detectable to high amounts [22]. Oral application of animals with radiolabeled PAs led to the disappearance of most of the radioactivity within 24 h, but small amounts remained detectable for many months in editable tissues, particularly liver. In other studies, none of the analyzed bovine or poultry meat or liver samples contained measurable amounts of PAs.

### 5.3. Plant Families Most Involved in PA Liver Injury

Liver injury by unsaturated PAs has preferentially been observed with exposure to the following plant families: Ariaceae (*Castilleja* spp.), Boraginaceae (*Heliotropium* spp. *Trichodesma* spp., *Symphytum* spp. such as Comfrey), Compositae (*Senecio spp*. like Bush teas, *Eupatorium* spp.), and Leguminosae (*Crotalaria* spp.) [24].

### 5.4. Products for Human Use Containing Unsaturated PAs Derived from Plants

#### 5.4.1. Bee Products

Most bee products, such as honey, bee pollen, propolis, and royal jelly, are consumed by humans in a belief in their health benefits, although evidence-based data in support of such beneficial properties are limited [26,27]. Better studied is the potential contamination of bee products with PAs; for example, by bees collecting material from flowering plants that contain PAs [28,29,30,31,32,33,34,35,36]. However, of theoretical interest only are these abundant reports, because they focus primarily on the amount of PAs in bee products rather than on the issue of whether or not even high amounts of PAs are potentially dangerous to consumers, e.g., causing liver injury in the form of HSOS.

Honey is known as a supersaturated sugar solution and is composed of various chemicals, such as phenolic acids, flavonoids, ascorbic acids, and carotenoids [27]. PAs have been detected in honey and other bee products originating from PA-containing plants [28,29,30,31]. Bees form pellets of pollen from flowers and store them in their beehive in special cells, while the transformed nectar continues its transformation to honey by losing water. As the second largest global producer and the largest global consumer of honey [28,32], Europe is privileged for studies on honey quality [32]. In a study from Poland, the PA content in local honey products was quantified using a gas chromatography-mass spectrometry method, and ranged from 1.0 to 20.2 μg/kg [28]. Additional results of PAs in honey have been reported from Italy (range from 0.6 to 17.6 μg/kg), Italy and Spain (up to 225 μg/kg), Germany and Austria (maximum 28.2 μg/kg), and Germany, Bulgaria, and Romania (ranging from 1 to 43 μg/kg), as compared with honey of Asian origin (ranging from 4.0 to 64.1 μg/kg) [28]. Although honey samples, positive for PAs, were in the majority, some samples showed a lack of PAs. The failure to detect PAs can be ascribed to the incapability of the plant visited by the bees to, itself, biosynthesize PAs, the growth of the flowering plant in a soil environment not contaminated with PAs, or by perfect production methods that prevent plant products from being contaminated with PAs derived from other nearby plants. Based on this single study [28] presenting low PA contents in honey, clearly highlighted as having been adapted from other studies, which was, in fact, a method-development report and not an overview of a significant number of honeys, the overall conclusion was that the estimated average dose should not cause a health problem and seemed likely to be safe [28]. However, other studies reported much higher amounts of PAs in honey [29,30,31]. For instance, in more than 2800 bulk honeys from Europe, the mean PA amount was 45 µg/kg, with a peak of 1087 µg/kg [29]. Similarly, in 48 local honeys from Ghana, PAs were found with a mean of 283 µg/kg and a maximum of 2639 µg/kg [30]. Finally, in 437 local honeys from Northern Germany, amounts of PAs were found with a mean of 73 µg/kg and a maximum of 3313 µg/kg [31]. None of these reports provided evidence for HSOS in consumers of honey with these large amounts of PA [29,30,31]. An internet search revealed no HSOS cases due to honey or other bee products, best explained by low PA consumption by humans, or unavailability of a thorough clinical or epidemiological search for individual patients at risk.

Bee pollen, a mixture of flower pollen, nectar and bee saliva, may contain PAs, based on an analysis from Italy using near-infrared spectroscopy methods or liquid chromatography coupled to tandem mass spectrometry [33]. In this study, using samples of bee pollen from Italy and various European countries, PAs were detected in a broad range of amounts, from 2 to 3356 μg/kg, with a mean of 382 ± 736 μg/kg (SD) and comprising 17 different PA types, again, with a broad range. The high variability of amount of PAs in bee pollen was ascribed to the fact that bees may collect pollen from plants containing PAs and from those not producing them [33]. It was also outlined that PA amount and composition in plants depends on the botanical taxon, geographical area of growing [33,36], and developmental stages of the plants [29]. In addition, PA synthesis in plants is influenced by various field conditions such as soil fertility, water availability, climate [33,37,38,39], and seasonal factors, such as time of harvest [36].

As another typical bee product, propolis or bee glue is a resinous mixture that bees produce by mixing saliva and beeswax with exudate gathered from tree buds, sap flows, or other botanical sources, to be used as a sealant for unwanted open spaces in the beehive [26,27]. Royal jelly, a viscous product from hypopharyngeal and mandibular gland secretion from the worker bees, is classified as special food preferentially consumed by the queen bee [26]. Based on samples collected in Germany, the Netherlands, Spain, France, Italy, and Spain, maximum PA contents were 48 μg/kg for propolis or royal jelly with a mean of 7.6 μg/kg; these values were low because propolis and royal jelly are produced independently from and outside of PA-producing plants and thus have substantially less as compared with amounts of PA in bee pollen, with a maximum PA of 1911 μg/kg and mean of 576 μg/kg [34].

#### 5.4.2. Vegetables, Including Spices

There is a long traditional use of PA-containing plant leaves as vegetables and spices, because most of such leave spiciness is likely due to PAs as ingredients, and these were not considered toxic for a long time because of a lack of analytical data and clinical awareness [22]. According to current knowledge and from study of the leaves of some plants, PAs are found in variable amounts in a variety of vegetables and spices, as presented by EFSA from samples in the Netherlands (Table 2) [23].

PA values are rather high in artichoke, fennel, and dandelion (Table 2) [23], similar to other herbs and spices like borage, oregano, and lovage with large amounts of unsaturated PAs of 3000 μg/kg [21], with borage showing levels of up to 31,101 μg/kg expressed in the dry product [23]. The reasons for these high levels are unknown [21,23] and thereby open for speculation, reflecting a high plant oxidative stress-induced synthesis rate of a common and/or a few selected PA compounds, PA acquisition via horizontal transfer from PA-containing plants, or some contamination of the plant product with parts from PA-containing plants. Of note, some plant products lack PAs (Table 2), either due to missing contamination or their inability to synthesize PAs, wherein the factors affecting this non-production remain to be explored. An unanswered question, also, concerns to what extent the PAs in plants are due to PA uptake from soil contaminated with PAs originating from nearby plants, known as horizontal transfer. To study this, a range of plants should be selected to be used as potential PA-acceptor plants, growing in soils contaminated with PAs. In the context of consumption by humans, a few leafy PA-containing plants—in particular, species of the *Borago* or *Symphytum*—are used in salads [22]. A caution is needed, here, because the leaves of the common weed *Senecio vulgaris* can be found together with salad leaves of similar appearance being sold in Germany.

There is also a long tradition in Germany, particularly in and around Frankfurt am Main, to consume the so called “Frankfurt green sauce” prepared from seven herbs: borage, burnet, chervil, chives, cress, parsley, and sorrel [22]. The leaves taste like fresh cucumber and have been found to contain a variety of PAs: intermedine, lycopsamine, amabiline, and supinine. PA plants, especially borage, are used as spices or ingredients for dishes in other European countries, also, known as sala verte, frittata di boragina, or as spice for cucumber salad [40]. There are no data available on how much of these PA products are consumed, nor on the amounts of PAs in these dishes that certainly show variations in their respective proportions of PAs contributed by the plants used to make them. Individually, for borage in its dry product high amounts of PAs of up to 31,101 μg/kg have been published [23].

#### 5.4.3. Bread and Grain

Bread may contain PAs if grain was used contaminated with PA-producing seeds that are co-harvested with grain [22,23]. Under normal conditions of good agricultural and manufacturing practices, attempts prevail to remove foreign seeds in grain including those containing PAs prior to milling [22]. As a result, large-scale intoxications seen in developing countries were not observed in developed countries [4]. However, a complete removal of PA-containing seeds is rarely achieved, especially in heavily contaminated grain or if the seeds were broken due to overripening, a special risk basically difficult to prevent unless PA-producing weeds are constantly removed from the soil mechanically prior to sewing out the grain [21,23]. Neglecting this procedure explains, in part, why, in grain, PAs are still detectable in amounts ranging from 23 to 320 μg/kg [23]. An alternative would be that grains such as wheat take up PAs from soil contaminated with them.

#### 5.4.4. Herbal Teas, Milk, and Dairy Products

Herbal teas are commonly prepared from leaves derived from special plants, which may be contaminated with unsaturated PAs or contain PAs synthesized by them. This explains why, in some herbal teas, PAs are detectable, if prepared from PA-containing plants like coltsfoot (*Tussilago farfara*), comfrey (*Symphytum officinale*), borage (*Borago officinalis*), climbing groundsel (*Senecio scandens*), and sunn hemp (*Crotalaria juncea*) [41]. For the purposes of evaluating whether PA extraction is different using intact leaves as compared with comminuted leaves, two types of herbal teas were prepared using boiling water, one from intact leaves and another one from comminuted leaves obtained from intact leaves, using a grinder. It seems that the extraction procedure of PAs from leaves may modify PA concentrations and is of low efficiency in intact leaves, because the total PA concentrations extracted from intact leaves were substantially lower, as compared with levels obtained from comminuted leaves, ranging from 30.7 to 845.4 μg/L for intact leaves and from 61.3 to 1120 μg/L for the comminuted leaves [41]. A substantial variability of PA concentrations was observed among the tested plants, with borage providing the highest PA levels. In another survey across Europe, considerably higher amounts of PAs were found in teas prepared from other plants, whereby leaves were used following homogenization and transfer to tea-infusion bags [34]. In one study, PAs in seven tested teas showed a maximum value of 4805 μg/L and mean values of 454 μg/L, and presented maximum PA values for black tea (4062 μg/L), green tea (3917 μg/L), rooibos tea (4805 μg/L), chamomile tea (1394 μg/L), peppermint tea (4401 μg/L), and mixed herbal tea (1929 μg/L). The homogenization of leaves may facilitate the liberation of PAs from plant structures and explain the high PA concentrations. Considering all conditions, the minimization risk is best achieved using intact leaves for tea preparation, but a better choice would be using leaves of plants containing low amounts of PAs. Green tea and black tea are, worldwide, the most commonly consumed beverages apart from water [42]. Consuming green or black tea prepared from PA-containing leaves of *Camellia sinensis* seems not to be a toxic PA risk to the liver, because epidemiology studies and clinical experience have not shown PA-HSOS in cohorts of patients consuming these teas, prepared by boiling and consumed in normal amounts. Only leaf extracts of green tea with high amounts of catechins are toxic to the liver, which is unrelated to PAs, because liver histology was exclusively of the hepatocellular or mixed type and never of the PA-related HSOS type [42].

In milk of cattle and after experiments with application of different PA containing plants to cows via a rumen fistula, the amounts of PAs were low, with 1.5 ± 1.2 μg/L in cow milk following the application of common groundsel [43]. In other studies, cow milk contained PAs in a range from 0.03 to 0.05 μg/L [37], or from 0.04 to 0.4 μg/L [44]. Comparing the PA profiles of the herbage plants the cows consumed with the milk they subsequently produced showed some qualitative and quantitative concordance for two out of three PA profiles. The difference for the third PA profile must have occurred along the transfer of PAs from herbs to milk by factors such as bacterial or enzymatic degradation, metabolic waste disposal, or reduced assimilation during digestive processes [45]. Fermented milk products like yoghurt, as well as cheese, had PA levels <LOD (limit of detection) [34]. As expected, however, PAs can well be detected in cheese produced from milk provided by cows experimentally receiving a mixture of PA-containing ragwort (*Jacobaea vulgaris*, syn. *Senecio jacobaea*) and narrow-leaved ragwort (*Senecio inaequidens*) via a rumen fistula. Under these conditions, the amount of PAs in cheese was 15 μg/kg as compared with 175 μg/L of the milk from which the cheese was produced [45]. The reduction of PA levels during the 6-week ripening of the cheese was explained by microbial degradation. As a result, qualified product management, including better agricultural practices, such as the early and continuous removal of PA-containing weeds, are required to reduce PA-containing weeds as part of the feed provided to dairy cows, and best achieved, finally, by keeping cows on or moving to grazing land free of PA-producing plants.

#### 5.4.5. Herbal Medicines

Modern and traditional herbal medicines commonly use selected herbal products as medicinal plants, some of which contain PAs, to treat patients with less serious ailments [22], although the efficacy for most indications is insufficiently reported due to the lack of positive results based on randomized controlled trials (RCTs) [46,47]. HILI, in the context of herbal medicine, remains a particular challenge as shown by multiple published liver injury cases [8,9,10,11,19]. Yet, HILI by TCM was rarely observed in a prospective study in Germany, due to having checked the quality of the herbal TCM medicines prior to use [48].

Medicinal plants containing PAs are described in Europe [22,49] and countries such as the USA and Canada [50], China [51], Mongolia, Nepal and Tibet [52], India [53], Sri Lanka [54], and Iran [55]. PA-producing medicinal plants belong to the plant families Apiaceae, Asteraceae, Boraginaceae, and Leguminosae, their plants produce a high variability of PA profiles, well evaluated for the past 30 years and perfectly listed with abundant references in a recent report [56], and now presented in modified form, including most of its listed references (Table 3).

A variety of analytical methods are available for detecting PAs in medicinal plants, as comprehensively outlined by Kopp et al. [56,122]. Apart from effective extraction procedures, selective and sensitive analytical techniques are essential. Considering the various analytical approaches focusing on selectivity and sensitivity, it seems that methods using liquid chromatography-mass spectrometry (LC-MS) are the most preferred, as they combine the physical separation properties of liquid chromatography with the mass analysis provided by mass spectrometry [56,122]. In general, the analysis of PAs in various matrices is a quite challenging task, as outlined in excellent reviews and reports from the last few years tackling analytical methods for PAs in certain feed and food [123,124,125,126].

First, a method for rapidly and accurately determining nine PAs with ultra-performance liquid chromatography–electrospray ionization-quadrupole-time-of-flight mass spectrometry (UPLC–ESI–Q-TOFMS) was developed and validated [123]. In addition, 70 PAs, their *N*-oxides precursors, and the characteristic fragment ions that are generated according to their chemical structure, were characterized. A method for the chemical profiling of alkaloids was also proposed, using the mass information obtained from the chromatograms of the tested sample. Lycopsamine, senecionine, senkirkine, and echimidine were identified in four potentially PA-containing plants and quantified by matching with authentic standards. Eight PAs and PANOs were also tentatively identified, using the mass data from the previously listed alkaloids. This approach will provide a database that can be used to instantly identify alkaloids in UPLC–ESI–Q-TOF MS botanical samples.

Second, a liquid chromatography tandem mass spectrometry (LC-MS/MS) was developed and used as a sensitive analytical method for determining 44 PAs in 18 tea samples from a local supermarket, and at least one PA/PA-*N*-oxide, in the 17 out of 18 samples under consideration, was detected [124]. Knowledge of the PA/PA-N-oxide composition was considered useful in searching for the botanical origin of the impurity, and the geographical region of the cultivation.

Third, a novel method was developed and optimized, which enables the determination of 33 PAs together with their *N*-oxides [125]. For the analysis of an aqueous-methanolic extract, reversed phase ultra-high-performance liquid chromatography and tandem mass spectrometry (RP-U-HPLC-MS/MS) was employed. The method was validated for frequently contaminated matrices of oregano and mixed herbal tea. As regards the achieved limits of quantification (LOQ), their values were in the range of 0.5–10 μg kg^−1^. The crucial problem encountered during method development, the co-elution of multiple groups of isomeric alkaloids, was overcome by subsequent sample separation in the second chromatographic system, with hydrophilic interaction liquid chromatography (HILIC), providing different separation selectivity. Lycopsamine, echinatine, and indicine (co-elution group 1) and *N*-oxides of indicine and intermedine (co-elution group 2) that could not be resolved on the commonly used RP column were possible to separate fully by using the HILIC system.

Fourth, an analytical workflow, including mass spectral library, generic sample preparation, chromatographic separation, and analysis by high-resolution mass spectrometry (HRMS) was developed to gain insight into the occurrence of plant toxins, mycotoxins and phytoestrogens in plant-based food [126]. This workflow was applied to 156 compounds, including 90 plant toxins, such as PAs, in plant-based protein ingredients, cereal and pseudo-cereal products. A mass-spectra library was built based on fragmentation spectra collected at 10 different collision energies, in both positive and negative ionisation modes, for each toxin. Emphasis was put on a generic QuEChERS-like sample preparation, followed by ultra-high-pressure liquid chromatography using an alkaline mobile phase, allowing the separation of more than 50 toxic PAs. HRMS acquisition is comprised of a full-scan event for toxins detection, followed by data-dependent MS2 for toxin identification against mass spectrum. The method’s performance was evaluated using fortified samples in terms of sensitivity, repeatability, reproducibility and recovery. All toxins were positively identified at levels ranging from 1 µg kg^−1^ to 100 µg kg^−1^. Such a workflow, using a generic, sensitive and selective multi-residue method, allows better insight into the occurrence of regulated and non-regulated toxins in plant-based foods, and to conduct safety evaluation and risk assessments when needed.

**Figure 2:**
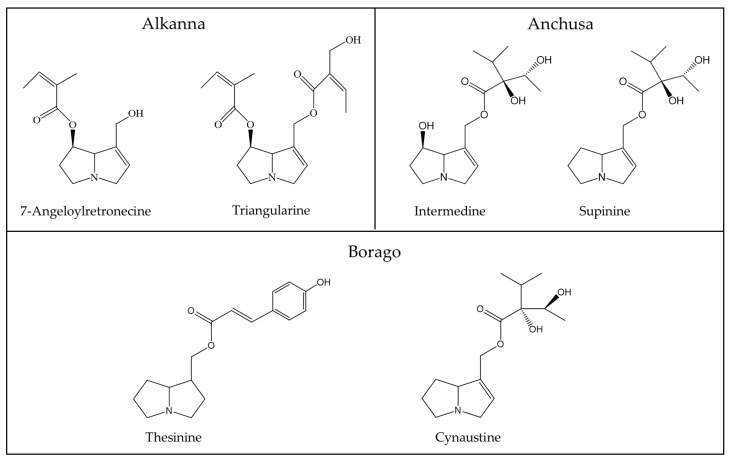
Selected pyrrolizidine alkaloids reported for the genera of *Alkanna*, *Anchusa*, and *Borago*. With the exemption of Thesinine, all other PAs are unsaturated and have a double bond between C1 and C2. The figure of the chemical structures was obtained from the open access report of Kopp et al. [56].

**Figure 3:**
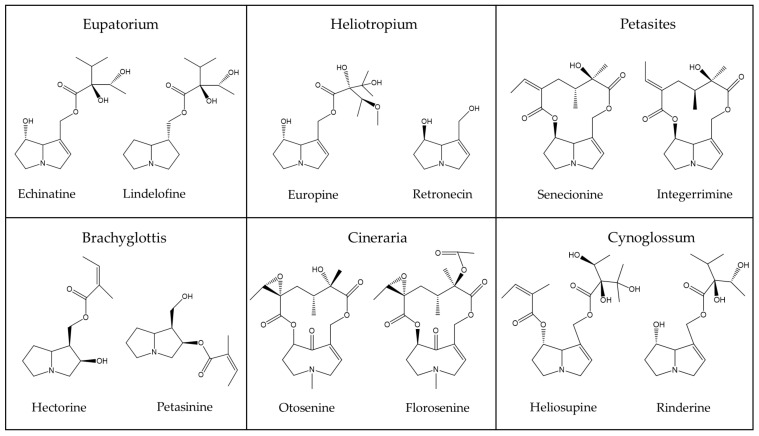
Examples of pyrrolizidine alkaloids reported for typical phytochemicals found in plants of the genera *Eupatorium*, *Heliotropium*, *Petasites*, *Brachyglottis*, *Cineraria*, and *Cynoglossum.* With the exemption of Lindelofine, all other PAs are unsaturated and have a double bond between C1 and C2. The figure of these chemical structures was obtained from the recent open access publication of Kopp et al. [56].

**Figure 4:**
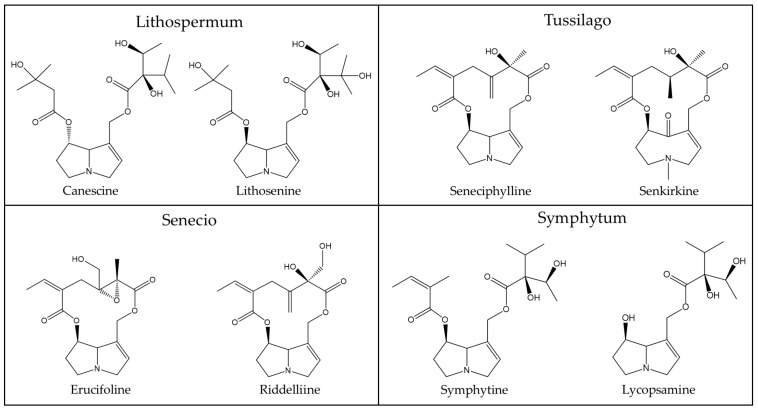
Examples of pyrrolizidine alkaloids inherent in plants of the genera *Lithospermum*, *Tussilago*, *Senecio*, and *Symphytum*. The figures with these chemical structures were obtained from the open access report of Kopp et al. [56].

In order to provide an overview on some typical PAs of phytopharmaceutical relevance as shown above (Table 3) and discussed by Kopp et al. [56], a few chemical structures are presented as examples for a general overview (Figure 2, Figure 3 and Figure 4) [56].

## 6. Interactions of PAs Released from Plants into a Water Environment

In a worst case scenario, humans with HSOS may have been exposed to PAs via drinking water contaminated with PAs derived from plants and released into the water environment. This was finally proposed, for instance, in an HSOS outbreak observed in Ethiopia, although confounding variables initially prevailed, because PA-containing herbs are used as medicines by a majority of the inhabitants in this country [68], and bread was found to be contaminated with PAs originating from the PA-producing *Ageratum conyzoides* spp. that grows widely in the grain fields as a weed [127]. These uncertainties called for additional studies to shed more light into the outbreak of the fatal liver disease of initially unidentified cause in Tahtay Koraro Woreda, Tigray region at the end of 2005 [128], leading to a series of publications [129,130,131,132,133]. The first study used an epidemiological study protocol with a combined descriptive and analytic design and a case-control approach based on a structured questionnaire [128]. Analyses focused on a case and control community within the Tsaeda Amba village and revealed differences in their water sources. Inhabitants of the affected community fetched their drinking water from an open, shallow and unprotected pond, Mai Habi-Tselam, whereas inhabitants of the unaffected community used other water sources in the same village [128], namely fresh water from river or unprotected spring [130]. In the meantime, the initial unidentified liver disease was diagnosed as HSOS due to PA toxicity [129], confirmed using experimental studies with laboratory animals following consumption of the suspected contaminated water [131], obtained from the unprotected well, in which the PA-containing plant *Ageratum* spp. abundantly thrived [132]. Additional information was provided in other publications [133,134,135]. Overall, these reports describe the challenges of establishing HSOS as a clear diagnosis and in searching for the origin of PAs. Based on these careful studies, it was shown for the first time that PA-containing plants can release PAs into water, likely via their roots, reaching into a nearby water source.

According to a recent study in Denmark, stream and seepage water in groundwater wells can be contaminated with PAs originating from *Petasites hybridus*, an invasive PA-producing plant from the Asteraceae family [136]. PA values of around 0.070 μg/L were found in stream water adjacent to plant fields of *Petasites hybridus* and a tenfold-increased concentration following intensive rain [136]. This was associated with PA values of up to 0.230 μg/L in seepage water from groundwater wells. Due to little awareness on PA contamination of water resources, regulatory definitions to limit PA concentrations in drinking water are not available [22,23,40,136]. The reports from Denmark did not indicate whether the drinking water contaminated with PAs may have caused HSOS among individuals consuming the contaminated water, although information was provided that senkirkine, senecionine and senecione *N*-oxide were the predominant 1,2-unsaturated PAs found in the water [136].

## 7. Uptake of PAs by Plants from Contaminated Water and Soil

On theoretical grounds, there are two conditions, whereby plants can acquire PAs: first, plants biosynthesize PAs using chemical intermediates as precursors and enzymes catalyzing the synthesis [24], and second, those plants that primarily cannot synthesize and therefore do not contain PAs may acquire PAs through soil or water contaminated with PAs [137,138,139,140,141,142]. Despite these promising data supporting the new concept of horizontal natural product transfer of PAs [137,138,139,140,141,142], a recently published study questioned the relevance of this phenomenon for PAs under field conditions [143], although many reports provided enough evidence that that horizontal tranfer is well functioning under field conditions for PAs [130,136] and various compounds such as heavy metals [144,145,146,147,148]. Indeed, the uptake of toxins by plants is known for heavy metals found to be severely contaminating soil, for instance, the vegetable *Ipomoea aquatica* consumed by nearby villagers [144]. Unintentional soil and water contamination may also explain the existence of toxins found in herbal medicines [13,145]. Instead, the intentional contamination of soil by heavy metals is provoked during growth of plants destined for medicinal herbs in Ayurveda [146,147,148] and anthroposophical medicine, for which this agricultural method is termed vegetabilization and said to enhance treatment efficacy [147,148]. Therefore and in line with mainstream opinion, plants can uptake both organic chemicals, such as PAs, and inorganic chemicals, like heavy metals.

With respect to PAs, much progress has been achieved within the last few years on the issue of their interactions with plants and the environment [137,138,139,140,141,142]. These conditions are real challenges, also recognized by the USP (United States Pharmacopeia) [149], and can help understand HSOS outbreaks from PAs in Ethiopia [68,127,128,129,130,131,132,133,134,135] and other subtropical and tropical countries [4]. The various facets of this fascinating PA concept, called horizontal natural product transfer, has been presented in publications since 2015 [137] und subsequently expanded [138,139,140,141,142], and is briefly summarized: (1) it was suggested that chemicals such as PAs may undergo translocation from a rotting PA-containing plant via soil into a nearby plant through uptake by its roots [137]; (2) the transfer of PAs was investigated using various herbs as acceptor plants, which had been mulched with dried plant material from the PA-containing *Senecio jacobaea* as providing plants, leading to high amounts of PAs in the nearby acceptor plants and confirming the horizontal transfer of PAs [142]; (3) the transfer occurs also from vital providing plants and is not limited to rotting plants, while the uptake by the roots of the acceptor plant proceeds through simple diffusion without an active transporter system, and, most importantly, the chemical imported may undergo modification in the acceptor plant [143]; (4) studies using co-cultures of the PA-containing *Senecio jacobaea* with parsley as an acceptor plant, which lacks its own PA biosynthesis, showed significant amounts of PAs in acceptor plants growing near the *Senecio* plants, of more than 200 μg/kg dry weight, previously synthesized by the *Senecio* donor plants [140]; and finally, (5) the uptake of PAs from the soil by the acceptor plant is variably determined by the rhizosphere pH, the concentration of protonated and unprotonated PAs in the soil, and the transpiration rate of the plant leaves [142].

**Figure 5:**
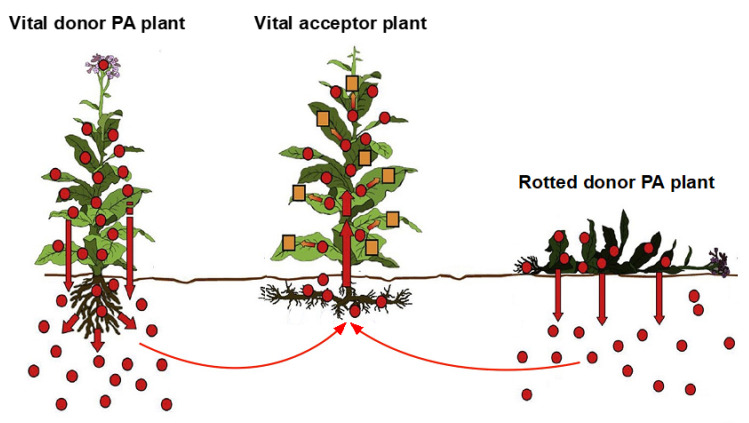
System of horizontal natural product transfer of pyrrolizidine alkaloids. Vital donor PA plants biosynthesize, in their leaves, PAs, which may reach the soil through various mechanisms. Including, preferentially, the rhizome and roots extruding from PAs. In addition, PAs, located in leaves of rotted or rotting donor PA plants, enter the soil. Assisted by surface water, PAs contaminating the soil arrive at the rhizome of roots of a vital acceptor plant, and, following their uptake, reach aerial parts of the plants, shown as small red dots, whereas some PAs may undergo metabolic modification, shown as small yellow squares. Abbreviation: PA, Pyrrolizidine alkaloid. This figure was modified and adapted from previous illustrations and suggestions of the group of Selmar [137,138,139,140,141,142].

The proposed system of horizontal natural product transfer has substantially enlarged our knowledge of PA interactions in plants acquiring PAs through contaminated soil, and allows for identifying mechanistic steps, with focus on plant roots and rhizomes responsible, for PA uptake [137,138,139,140,141,142], and containing PAs in addition to aerial plant parts [56,133,150]. According to published evidence and illustrations [137,138,139,140,141,142], it seems that most of vital plants can act as acceptor plants, receiving, through their roots and rhizomes, PAs released from vital donor-PA plants or rotted donor-PA plants (Figure 5).

Apart from the PA issue, plant biology studies on allelopathy, like those of Selmar and his group are relevant for PA plants and neighboring plants [137,138,139,140,141,142], but, if expanded, they could provide additional information on commonly recommended crop rotation in agriculture, gardening rotation practices for some flowering plants, and the co-cultivation of certain vegetables [137]. More details would be appreciated if these physiological and pathological studies also could focus on interactions between Glyphosate (Roundup^®^)-derived herbicidal compounds and weeds, in addition to the recently described cordycepin, isolated from the edible *Cordyceps militaris,* with its newly discovered, highly effective herbicidal properties and potentially plant-based novel alternative to Glyphosate [151].

## 8. Principles of Plant PA Biosynthesis

PA biosynthesis and the pattern of PA-diversified profiles were the subject of a variety of publications [24,56,68,152,153]. They covered recent advances of biological PA synthesis and also basic results, reported in earlier publications. In addition, the chemical structures of individual PAs were presented in many reports [24,37,56,152,153]. Of interest were also discussions on PAs as secondary plant metabolites interacting with primary plant metabolites [37,152], the abiotic and biotic oxidative stress modifying environmental conditions of growing [39,149,150,151,152,153,154,155], and diurnal variation influencing PA biosynthesis [39].

### 8.1. PAs as Secondary Metabolites

Plants produce compounds that are commonly classified as primary and secondary plant metabolites [37,152]. Among the primary metabolites (PMs) are chemicals such as lipids, proteins, and carbohydrates, directly involved in maintaining plant structure, development, and growth [152]. As opposed, PAs, as members of the large group of plant secondary metabolites (SMs), are assumed to be built by plants against herbivores [37]. These conditions qualify PAs as typical SMs [152]. They are commonly repellent for generalist herbivores, which do not discriminate between foods with high and low amounts of PAs, while particularly attractive to specialists, which are qualified for this specific discrimination [154]. Most interestingly, in PA plant interactions may occur between PMs and PAs, resulting in increased or decreased production of PAs qualified as SMs [37]. PAs themselves are presumably unessential for growth and survival of plants and not directly involved in maintaining plants in good conditions. However, PAs may contribute to plant growth and survival by protection of plants to be damaged from herbovorous attacts.

### 8.2. Synthesis of PAs and Plant Reactive Oxygen Species with Abiotic and Biotic Oxidative Stress

The enzymatic synthesis of PAs in the plants has to be seen in the context of oxidative stress initiated by reactive oxygen species (ROS), both important conditions along with abiotic or biotic plant stress [155,156,157,158,159,160,161]. Biotic stress results from so-called pathogen attacks by other living organisms, among these are insects and larger grazing animals, parasites, bacteria, viruses, and fungi. Whereas, abiotic stress has an environmental background and is commonly initiated by conditions or factors such as heavy UV radiation, draft, wounding, or soil contaminated with salts or heavy metals [39,161,162,163,164]. In general, plant stress damages the integrity of the plant and is often the cause of limited plant-product quality. The production of SMs is closely related to the growth conditions of plants, a view based on increasing evidence implicating plant oxidative stress in the synthesis of SMs [156]. Characteristic features of SMs synthesized by a plant depend on the species, genotype, physiology, developmental stage, and environmental factors during growth.

### 8.3. PA Synthesis, Plant Circadian Clock System, and Seasonal Variation

Plant functions and integrity commonly depend on a variety of specific variables, such as molecular-based genetic plant circadian clock system syn. diurnal cycle [145,161,162] and seasonal variation [145,162], conditions to be considered for PA-containing plants [162]. First, studies on diurnal cycles implicated in PA synthesis showed that the leaf metabolome was affected by the time of harvesting, with PAs and succinic acid accumulating in the morning (10:00 h), while sugars and formic acid accumulated towards the evening (19:00 h), substantiating the existence of a diurnal cycle and suggesting that harvest time is an important factor in metabolomics results [163]. Second, PA synthesis is dependent on developmental stage and thus on the season of harvest, shown in a study from Italy presenting an average PA content of plants of 0.33% of dry weight over the growing season, in which the highest PA levels, of around 1%, were found in the young sprouts and flower heads [164], whereas another study showed increased total amounts of PAs during plant development, correlating with an increase in biomass at nearly constant PA concentrations [39]. More specifically, the highest PA concentrations ranged from 3.2 to 6.6 g/kg dry weight and were found in plants from the beginning of July until the middle of October when the rainy season begins [165], findings that are in line with increased PA concentrations in plants under drought conditions [166,167]. PA production in plants was reduced under the use of nutrients such as NBK (nitrogen, phosphorus, and potassium) fertilizers, which are complete fertilizers [168].

### 8.4. Site of PA Biosynthesis in Plants

Sites of synthesis, translocation and accumulation of PA *N*-oxides were studied using ^14^C-labelled PA precursors provided to *Senecio vulgaris* plants via the root system [169]. These precursors were rapidly incorporated into PA senecionine N-oxides, most were translocated into the shoot and the inflorescences, the major sites of PA accumulation, suggesting that the roots are the primary site of PA synthesis. Additional studies on transport details have revealed that the PA *N*-oxides do not simply follow the transpiration stream but are specifically channeled to target tissues, such as epidermal stem tissue and flower heads [169]. The different steps of PA biosynthesis were outlined in detail more recently [37].

### 8.5. The Enzymatic Steps of PA Synthesis in Plants

PAs are synthesized in plant roots from various precursors such as ornithine [152], arginine, putrescine, and spermidine [169], with homospermidine synthase as the key catalyzing enzyme [37,170]. The chemical structure of PAs synthesized in plants and used as herbal medicines is quite variable, as shown for a few examples (Figure 2, Figure 3 and Figure 4). The 1,2-unsaturated PAs exert their potency of liver injury in humans, not a priori, but only after their metabolic bioactivation in the intestinal tract by means of the gut microbiome and the liver [171], through reverting the enzymatic desaturation, initially achieved by the plants, now carried out through dehydrogenation of the double bond between C1 and C2 (Figure 1) [37].

**Figure 6:**
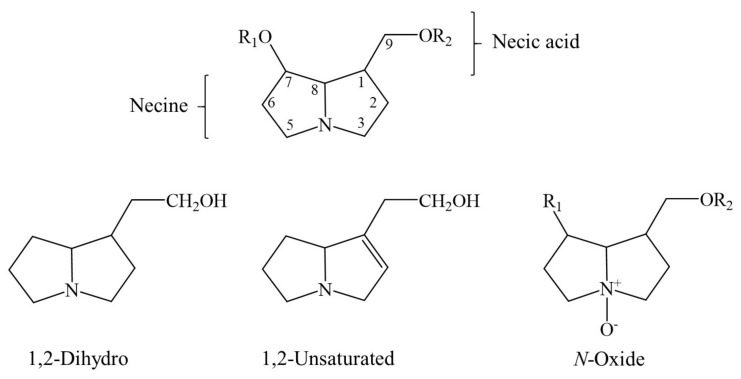
Structure of a PA and its different forms. R_1_ and R_2_ correspond to different necic acids. Abbreviation: PA, Pyrrolizidine alkaloid. The figure was obtained from the open access report of Moreira et al. [152].

The structural diversity of PAs is overwhelming and based on various factors [37,152,172,173,174,175,176]. PAs rarely occur in the free form of a pyrrolizidine base, but usually present as variable esters (mono-, di- or macrocyclic diesters), formed by a necine base, necine in short, and by one or several necic acids (mono- or dicarboxylic aliphatic acids), which finally determine the structural PA diversity [152]. PAs are found in the variable forms of tertiary bases of 1,2-dihydro and 1,2-unsaturated or *N*-oxides (Figure 6) [152].

Amino alcohols, or necines, are derived from pyrrolizidine [152]. Pyrrolizidine’s core, comprizing two saturated five-membered rings with a nitrogen atom between them, sometimes shows a double bond between C1 and C2 (Figure 5), with the potential property of enhanced toxicity as unsaturated PA. They also can have a single alcohol at the C1 position, another alcohol at C7 (dihydroxylated) and, less often, a third alcohol at C2 or C6 (tri-hydroxylated). Esterification is possible at C7 and/or C9 [152]. According to the structure of the necine base, 1,2-unsaturated PAs may be sorted into three main groups, the retronecine, heliotridine, or otonecine type, whereas saturated PAs are of the platynecine type [152].

Although much work has been done on identifying the various PA compounds and their intermediates [24,37,152,171,172], limited information is available on their biosynthesis in plants [152]. In particular, there is a knowledge gap of the enzymatic steps leading to PA unsaturation between C1 and C2, with many steps listed with a question mark [37]. Highly appreciated under clinical aspects, both toxic and non-toxic PA compounds can be distinguished and quantified by the analytical LC-MS method [173].

## 9. The Role of Cytochrome P450 in Metabolizing and Toxifying Unsaturated PAs

Cytochrome P450 (CYP) plays a pivotal role in the metabolic degradation of 1,2-unsaturated PAs [37,171,172]. After oral uptake, PAs reach the intestinal tract and, from there, the liver of the consumer. Although CYP, with its various isoforms, is present in both locations in humans and animals, it is found much more in the liver, as compared with the intestinal tract [1,2,3,177,178,179,180]. Most importantly and specifically in human hepatocytes, 1,2-unsatured PAs are degraded by several CYP isoforms [172]. Whereas less active CYP isoforms include CYP 2B6, CYP 2D6, CYP 2C9, CYP 2C19, and CYP 2E1, the clinical focus is on the biologically most active CYP isoforms CYP 2A6, CYP 3A4, and CYP 3A5 that show striking differences in their substrate specificities [172], with the possible consequence that the degree of liver toxicity may be variable depending on the PA type to be degraded and the specific CYP isoform involved in the degradation of one of these PA types. Details of the PA types metabolized by one of the most active CYP isoforms are listed (Table 4) [172].

Unsaturated PAs are toxic, provided their double bond between C1 and C2 (Figure 1) is metabolically removed through transhydrogenation [37] via the so called dehydrogenation pathway [178]. The reaction is catalyzed by CYP [37,172,178], a hemeprotein with a broad substrate specificity, metabolizing a variety of exogenous compounds, such as other phytochemicals, drugs, ethanol, and aliphatic halogenated hydrocarbons, such as carbon tetrachloride, and located in the endoplasmic reticulum of the liver cell [1,2,3,6,20,177,178,179,180,181,182,183,184]. The metabolism of unsaturated PAs occurs in the hepatocytes [172] and not in the LSECs that do not express active CYP isoforms like CYP 3A4 [185], essential for PA degradation (Table 4) [172]. Instead, LSECs merely express the CYP 1B1 and CYP 2E1 isoforms [185,186,187], both lack relevance for enzymatic PA degradation [172]. LSECs with their CYP 2E1 metabolize ethanol [187], and CYP 1B1 impacts the angiogenic and inflammatory properties of LSECs [185]. Most interestingly, LSECs can metabolize unsaturated PAs only in a novel CYP 3A4 transduced human LSEC model, which can be used for screening liver injury by PAs [187].

The endoplasmic reticulum, as the ultrastructural organelle of the hepatocytes harboring the CYP isoforms relevant for PA degradation (Table 4), is visible by electron microscopy and corresponds to the microsomal fraction of the biochemists. The microsomal metabolism of exogenous compounds requires not only CYPs, but also phospholipids, as structural constituents of and present in the membranes of the endoplasmic reticulum, as shown by reconstitution experiments [188,189,190] and perfectly summarized in a recent report [191]. The CYP-dependent reaction also requires molecular oxygen and NADPH + H^+^ (reduced nicotinamide adenine dinucleotide phosphate), which provides reducing equivalents for the NADPH CYP reductase, another obligatory constituent of the NADPH dependent CYP reaction [177,183].

**Figure 7:**
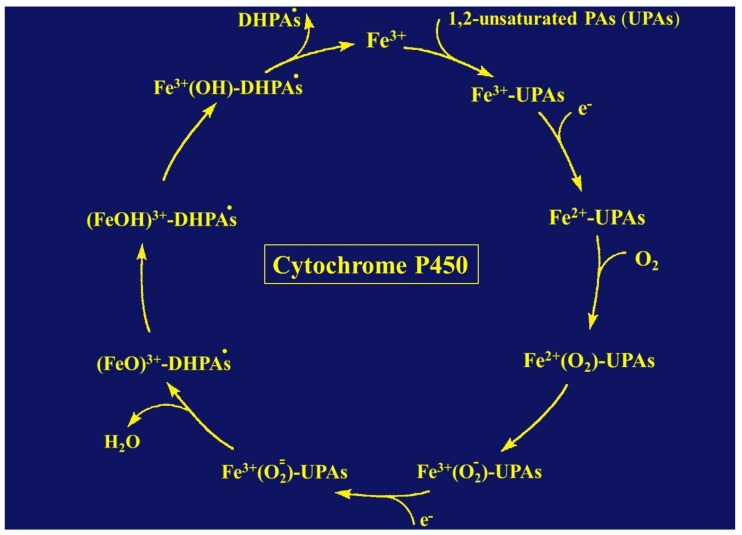
Proposed catalytic cytochrome P450 cycle implicated in the toxification of 1,2-unsaturated PAs. In analogy to drugs, ethanol and other exogenous compounds, the 1,2-unsaturated PAs (UPAs) enter the catalytic cytochrome P450 cycle as substrate to be metabolized, shown on top of the cycle right side. After several steps, the metabolized UPA leaves the cycle as toxic radical DHPA•, whereby the 1,2-unsaturated PA loses its double bond and changes to pyrrole protein adducts. CYP stands for its various isoforms. As a reminder, regarding cytochrome P450, the term “P450” was used to describe a “pigment” with an absorption maximum at 450 nm within the ferrous-carbon monoxide complex of CYP in rat liver microsomes [191]. The figure was modified and retrieved from an open access report of Teschke and Danan [184].

The NADPH + H^+^ is converted to NADP^+^ through the NADPH CYP reductase in its oxidized form that becomes reduced, while the reduced form of NADPH CYP reductase in turn converts the oxidized CYP to its reduced form. Whenever exogenous compounds like 1,2-unsaturated PAs enter the catalytic CYP cycle to be oxidized, they first must bind to the Fe^3+^ of the oxidized CYP (Figure 7) [182,183,184].

**Figure 8:**
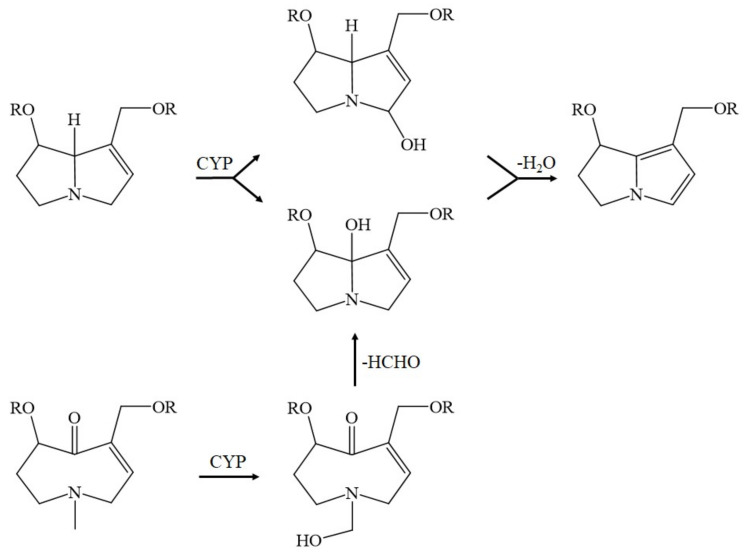
Interactions of CYP with some unsaturated PA forms. CYP modifies the chemical structure of some 1,2-unsaturated PAs, leading to the removal of their specific unsaturation between C1 and C2. Abbreviations: CYP, cytochrome P450; PA, pyrrolizidine alkaloid. The figure was taken from the open access report of Schramm et al. [37].

More specifically, the first electron is provided to CYP by NADPH + H^+^ via the NADPH CYP reductase and the reduced form of CYP with Fe^2+^ is generated, which finally becomes oxidized again after splitting off the oxidized substrate. CYP is then again free for the next substrate to be oxidized (Figure 7) [184]. Through introduction of molecular oxygen, a multi-compound reactive complex emerges, facilitated by inclusion of another electron that, commonly, is provided through the NADPH CYP reductase or a similar but NADPH-independent reductase. Under normal conditions, such as drug metabolism leading to drug oxidation, this CYP-dependent enzymatic process proceeds smoothly, especially in drug metabolism, but occasionally, and most likely, in connection with the metabolism of 1,2-unsaturated PAS, much ROS is generated from incomplete split of oxygen (Figure 7), leading to liver injury, but CYP concomitantly modifies the chemical structure of 1,2-unsaturated PA types [192], as shown for some examples (Figure 8) [37].

**Figure 9:**
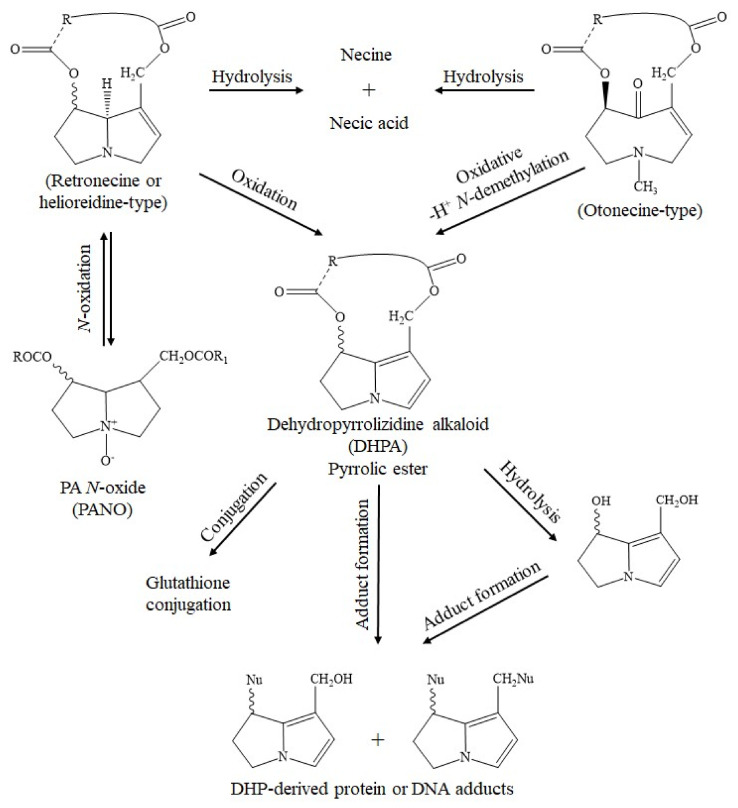
The fate of 1,2-unsaturated PAs to adduct formation. Different steps are delineated leading from 1,2-unsaturated PAs to their adduct formation with proteins and DNA. DHPA is partially detoxified through conjugation with glutathione. Abbreviations: DHP, dehydronecine pyrrolizidine; DHPA, dehydropyrrolizidine alkaloid; PA, pyrrolizidine alkaloid. The figure was retrieved from the open access report of Schramm et al. [37].

In detail, the retronecine-, heliotridine-, and otonecine-types of PAs lose their double bond between C1 and C2 and receive other conformational modifications at other places. The resulting dehydropyrrolizidine alkaloid (DHPA) or dehydronecinepyrrolizidine (DHP) are parts of the adduct formation with proteins and DNA (Figure 9) [37].

It seems that PA toxicity of extrahepatic organs is triggered by PA-related events in the liver cells, as shown at least in experimental PA studies on lung injury, which depends on the metabolism by hepatic CYPs and the blood transport of reactive metabolites [175]. However, it is conceivable that parts of the DHPA and DHP in the liver remain free of any adduct attached, leave the liver cell, are taken up by neighboring LSECs, or enter the systemic circulation before they dock with other cellular constituents, including DNA at organs outside of the liver.

## 10. Overview of the Mechanistic Molecular Aspects of 1,2-Unsaturated PAs in Clinical Toxicity

Recent in silico studies have revealed the hydroxylation of their necine base at C3 or C8 of the heliotrine- and retronecine-type PAs, or at the *N* atom of the methyl substituent of the otonecine-type PAs, but this step is only a defined precursor step of the formation pathway of toxic DHPAs and does not cause the toxic potential of 1,2-unsaturated PAs [178]. A critical question remains—what really does happen in the liver cell, at the site of CYP, with the double bonds of the 1,2-unsaturateted PAs prone for DHPA-derived and DHPZ-derived major disturbances of cellular integrity? The important observations and discussions quoted above led us to the view that a major role for toxicity by unsaturated PAs has to be attributed to ROS, produced during PA degradation via CYP leading to the generation of toxic by-products as radicals such as single radical **^1^**O**_2_**, superoxide radical HO•_2_, hydrogen peroxide H_2_O_2_, hydroxyl radical HO•, alkoxyl radical RO•, and peroxyl radical ROO•, conditions similar to those described for liver injury by ethanol [12,13,14,177] and, likely, also drugs [179,180,181,182] or carbon tetrachloride [20]. These radicals are toxic to membrane structures and the constituents of hepatic microsomes, with their proteins and phospholipids that undergo peroxidative processes. In addition, injury may involve liver mitochondria, because they also contain CYP, with specific pathways to reduce this catalytic CYP cycle [191]. It appears that many mechanistic molecular steps may trigger liver injury by unsaturated PAs upon their metabolic conversion [192,193]. Questions remain as to their potential role in the etiology of cancers, pulmonary hypertension, and congenital anomalies [194] and on the exact molecular basis through which unsaturated PAs can exert not only their hepatotoxic, but also their genotoxic, cytotoxic, neurotoxic, and tumorigenic properties [37]. Similarly, stimulating and partially understood are the clinical aspects of liver injury by 1,2-unsaturated PAs.

## 11. Clinical Specifics of HSOS Caused by 1,2-Unsaturated PAs

General agreement exists that the use of some plants or herbal medicine, whether they contain 1,2-unsaturated PAs or not, may create health problems, including liver injury, termed globally as herb-induced liver injury (HILI) [8,42,48,145,162,195,196,197]. However, liver injury in connection with the use of plants containing 1,2-unsaturated PAs is a special disease entity, further specified as HSOS [6,198,199,200], replacing the previous term “HVOD (hepatic veno-occlusive disease)” with “HSOS” upon suggestion by DeLeve et al. [199]. The note of a syndrome implies a complex, multifaceted disease, with focus on an obstruction type of the injury, related to the liver sinusoidal endothelial cells, the LSECs.

### 11.1. Definition of HSOS

HSOS, caused by 1,2-unsaturated PAs, PA-HSOS in short, has to be differentiated from other HSOS disease entities, such as due to haematopoetic stem cell transplantation, HSCT-HSOS in short [6,200]. Different diagnostic criteria are available for each entity, the Nanjing criteria for PA-HSOS [6,200], and the modified Seattle, or the Baltimore criteria for HSCT-HSOS [200].

According to the Nanjing criteria, the diagnosis of PA-HSOS can be established, if a definite history of ingesting a PA-containing plant is available and other causes of the liver injury are excluded, and if the following three items are met: (1) abdominal distention and/or pain in the hepatic region, hepatomegaly, and ascites; (2) elevation of serum total bilirubin or abnormal liver function testing; and (3) typical features of enhanced CT (Computed Tomography) and MRI (Magnetic Resonance Imaging) [200]. Care should be taken, for differential diagnoses including Budd Chiari syndrome, DILI, alcoholic hepatitis, and decompensated cirrhosis, in rare cases. Notably, among the Nanjing criteria there are missing threshold definitions of bilirubin and LTs. Not part of the Nanjing diagnostic criteria [200] was, for unknown reasons, a robust causality assessment method (CAM), like the updated RUCAM [201], also not included in the subsequent report on the validation of the Nanjing criteria, which showed a sensitivity of 95.35% and a specificity of 100% [202]. As the HSOS cases did not receive a valid CAM, the absence of RUCAM as the diagnostic gold standard remains a critical and major shortcoming, considering also that its application has strongly been recommended as part of the guidelines by the Branch Committee for Hepatobiliary Diseases and Branch Committee for Chinese Patent Medicines, China Association of Chinese Medicine for HILI cases [203]. As a consequence, the Nanjing criteria should be re-validated using PA-HSOS cases, assessed for causality using the updated RUCAM with causality gradings for PAs of probable or highly probable.

### 11.2. Classification of PA-HSOS

In line with the classification of DILI, HILI commonly emerges as two different forms, the idiosyncratic and the intrinsic form [19,197,201]. Accordingly, the idiosyncratic type of HILI develops unexpectedly at recommended doses of the herbal medicine or product, has a long and variable latency period, a low incidence in humans occurring in a few individuals due to their genetic condition, and is not reproducible in animal models. Conversely, the intrinsic type of HILI is dose-dependent, occurs in any individual independently of any genetic predisposition, has a short and consistent latency period, and is reproducible in animal models. PA-HSOS clearly belongs to the large group of the typical intrinsic liver injury type rather than the idiosyncratic type of liver injury. Clinical and experimental studies suggest that PA-HSOS is dependent on the daily dose of PAs, the duration of their intake, and the cumulative dose [3,20,21,22,23,24,174,204,205,206,207].

The reproducibility of PA-HSOS in animals has the advantage that results from animal studies can be translated to humans, and data of many mechanistic studies in animals can explain the individual steps leading to HSOS in humans. In addition, animal studies are helpful in cases of plant misidentification, a health threat for consumers and a clinical challenge for physicians to correctly diagnose the liver injury and find its cause. For instance, 41 cases of HSOS in China were published, causally attributed by error to the TCM (Traditional Chinese Medicine) plant Jing Tian San Qi (*Sedum aizoon*, syn. Stonecrop). In fact, *Sedum aizoon* does not contain any unsaturated PAs as ingredients, and its application to experimental animals did not result in the development of HSOS [208]. Consequently, the HSOS cases, as reported, must have been caused by PAs in connection with the use of another plant containing PAs such as *Gynura segetum* [209]. Similar to these cases is another liver injury case from Hong Kong, with HSOS that was also erroneously ascribed, initially, to *Sedum aizoon*, but turned out to have been due to the TCM plant Shan Chi (*Gynura segetum*) [209]. The name and appearance of *Sedum aizoon* are similar to *Gynura segetum*, but botanical differentiation was finally considered possible for experts [209]. At the end, studies comparing both plants provided clear evidence for *Gynura segetum* as then culprit for additional cases of HSOS, instead of *Sedum aizoon*. Respective studies in rats showed that *Gynura segetum* contains PAs and may cause experimental HSOS as confirmed by liver histology; conversely, toxic 1,2-unsaturated PAs were not found in *Sedum aizoon* [209]. In an earlier study, a model of experimental HSOS was established by PAs derived from a plant described erroneously as *Sedum aizoon* [210], which again does not contain PAs [209,210,211,212]. This suggests that the described experimental model [210] was due to the action of phytochemicals derived a plant, which contains PAs, most likely *Gynura segetum* [209,210,211,212], rather than *Sedum aizoon* that lacks PAs [211]. Based on these considerations, there is evidence for a hepatotoxic potential role of Jing Tian San Qi involved in other cases of plant misidentification. In two Chinese women, HSOS emerged, which was induced by PAs of the TCM plant *Gynura segetum* (syn. Ju Shan Qi, Ju Ye San Qi, San Qi Cao, Shan Chi) [213]. Additional six cases were suspected earlier [214,215]; in at least four of these, the culprit was the PA-containing plant *Heliotropium lasiocarpum* rather than *Gynura segetum* [216].

### 11.3. Epidemiology

Worldwide systematic PA-HSOS epidemiology data on incidence and prevalence details are difficult to retrieve, despite the common occurrence and use of plants containing PAs [20,21,22,23,24,37,40,56,149,174,198,199,200,201,202]. There are, of course, reports of single PA-HSOS cases published throughout the world, and PA-HSOS clusters with many cases reported preferentially in tropical and sub-tropical regions. As it stands, the lack of valid epidemiology results can be traced back to insufficient diagnostic approaches overlooking existing overt HSOS cases, low grades of clinical HSOS features, and the low awareness of this type of liver injury. In general, frequency of published PA-HSOS cases is low in Western countries and can be high in some selected tropical regions.

### 11.4. Clinical Characteristics

Patients with initial stages of PA-HSOS commonly present with unspecific features found also with other diseases and thus being of little diagnostic significance. Early symptoms may include minor abdominal pains, vomiting or nausea, and asthenia, they are too vague to help suspect the liver injury. Of diagnostic value is the fact that, despite loss of appetite and low caloric intake, the patients with PA-HSOS suffer from concomitant weigh increase due to fluid accumulation as edema throughout the body, including pleural effusion and ascites of the abdominal cavity. Nevertheless, preferentially sporadic HSOS cases often remain undetected. With disease progression, key features now include severe abdominal pains and rapid developing ascites, while jaundice may be present or not, these representing the tip of the iceberg. The variability of features may be explained by differences in the nutritional status, pre-existing disease, and amount and type of the ingested PA. Medical history regarding consumption of products potentially contaminated with plants containing PAs is primarily justified, although rarely expedient. Diagnostic conditions are generally better if clusters of HSOS cases emerge facilitating early suspicion.

Based on a rather homogenous, large study of 116 patients with HSOS caused by *Gynura segetum* as a single plant containing PAs, defined clinical characteristics can be found. Regretfully, the PA-HSOS cases were not assessed for causality using RUCAM, conditions that limit the quality of the published data. Nevertheless, the leading clinical features were from top to down: ascites, hepatomegaly, and jaundice (Table 5) [211].

### 11.5. Routine Laboratory Data

Serum activities of ALT (alanine aminotransferase) or AST (aspartate aminotransferase) were increased only in some, but not all, of the HSOS patients shown in a single study as example (Table 5) [211], a crucial point of diagnostic accuracy, because normal ALT or AST values do not exclude PA-HSOS, requiring caution, as PA-HSOS may easily be overlooked. In addition, actual data were not provided for the case series and five additional, otherwise well-documented cases with causality assessment using RUCAM [206]. However, subsequent perfect studies documented for RUCAM-based PA-HSOS cases of actual results of liver tests (LTs), such as ALT (243.4 ± 60.0 U/L) or AST (259.9 ± 62.7 U/L) [212], in line with other reports, as summarized recently [6]. LTs alone were obviously not the ideal screening parameters to detect PA-HSOS. Indeed, virtually all of the PA-HSOS cohort had ascites and thereby a late stage of HSOS, but part of the patients had no elevated values (Table 5) [211]. ALP (Alkaline phosphatase), and bilirubin was also of no additional diagnostic value [6].

### 11.6. Specific Diagnostic Biomarkers

Much progress was achieved with the establishment of diagnostic biomarkers using blood pyrrole-protein adducts (PPAs) [211,212,217,218,219,220,221,222,223,224], highly appreciated and discussed among the scientific community [3,4,5,6,37,183,197,198,225,226]. These specific biomarkers provide a prognostic index [217] but primarily help diagnose PA-HSOS, are mechanism-based, resulting from reactive metabolites generated during the degradation of unsaturated PAs and due to covalent binding to DNA, albumin and other proteins [211,212,217,218,219,220,221,222,223,224], as illustrated (Figure 9) [37]. The formed PPAs leave the injured hepatocytes and enter the systemic circulation, ready to be determined in the blood of patients with PA-HSOS. The long persistence of PPAs following PA intoxication is remarkable and of clinical importance [224]. Finally, as feasible potential biomarkers of PA exposure were other parameters tentatively promoted, including pyrrole-hemoglobin adducts [227] and blood microRNAs [223] requiring additional comparative studies with inclusion of PPAs.

### 11.7. Imaging Features

Diagnostic options include ultrasonography, Doppler ultrasound, computed tomography (CT), magnetic resonance imaging (MRI), and digital subtraction angiography [6,211,228,229,230]. These technical approaches should be considered as complementary methods, in addition to established criteria of PA-HSOS and the use of PPAs as diagnostic aid. Ultrasound is the most cost-effective method in the diagnosis of PA-HSOS, followed by Doppler ultrasound [6]. The combination of both methods will help search for hepatomegaly, reduced portal vein flow velocity, and various alterations of liver arteries and veins in form of stenosis, likely due to impression triggered by the large liver.

Partially confirming results by ultrasonography and Doppler sonography, typical signs obtained by CT are ascites, liver enlargement syn. hepatomegaly, and thickening of the gallbladder wall, while splenomegaly is a rare finding. MRI provides different information with focus on liver hypointensity or hypoattenuation during the hepatobiliary phase and enhancement around the portal veins during the portal venous phase [6]. For specific questions regarding suspected alterations of vessels the use of digital subtraction angiography may be useful, if therapeutic consequences are to be expected.

### 11.8. Liver Histology

Previous invasive diagnostic approaches, like biopsies of the liver in patients with suspected PA-HSOS, were helpful and clinically indicated to assist define the liver injury, using specific typical histological features to confirm the diagnosis [211] Yet, today, whether liver histology is still needed for the diagnosis to be considered as diagnostic gold standard by Yang et al. [6] is difficult to reconcile, and lacks support in face of PPAs as gold standard, now having been available for several years. So we do not advocate liver histology as an essential diagnostic method, but rather suggest to establish the diagnosis using the Nanjing criteria [200] and non-invasive, resources saving approaches like PPAs [211,212,217,218,219,220,221,222]. In reality, liver biopsy is now rarely done. For instance, in a recent study most patients with PA-HSOS did not receive percutaneous liver biopsy because of ascites, thrombocytopenia, and coagulation problems [231]. In addition, transjugular liver biopsy was performed in a few patients.

Previous results on liver histology allow for summarizing some important features [6,211,229]: (1) derangement of the liver architecture [211]: (2) endothelial swelling [212]; (3) extension and congestion of hepatic sinusoids [211,229] preferentially in zone 3 [229]; (4) connection of sinusoids to blood pools [211] and extravasation of erythrocytes into the space of Disse [229]; (5) fibrosis preferentially around the blood sinusoids close to the central vein [211]; (6) deposition of hemosiderin due to hemolysis [229]; (7) loss of hepatocytes around the central vein [211] and hepatocellular necrosis [229]; and (8) accumulation of macrophages and infiltration of inflammatory cells [229]. Of note, RUCAM based cases were used in the two reports [211,229], providing some degree of certainty that the diagnosis of PA-HSOS was correct.

Although light microcopy provided some interesting findings, data obtained by electron microscopy might provide additional insight information. An electron microscopy study in Jamaican children with PA-HSOS showed hepatocyte alterations, inluding dilatation of the bile canaliculi, abundant glycogen, nuclear invagination, and naked hepatocytes with a reduction of microvilli [204]. In a mouse PA-HSOS model, sinusoidal injury occurs in the early stage of experimental PA-HSOS, associated with damage to the fenestration of LSECs, which forces LSECs to transfigure into a disc-like shape, and is accompanied with the loss of LSECs by the degradation of the extracellular matrix [232]. Scanning electron microscopy images showed dilatation of sinusoids, enlarged and damaged fenestrae, and severe congestion in the liver of the model mouse. These results suggested that sinusoidal injury happened in the model group, similar to human PA-HSOS, but the focus of this study was not on hepatocytes and their possible subcellular alterations. In a rat model of PA-HSOS using monocrotaline published by DeLeve et al. [233], transmission and scanning electron microscopy showed early manifestation in the form of progressive injury to the sinusoidal wall, with the loss of sinusoidal lining cells, sinusoidal hemorrhage, and damage to the central vein epithelium, followed by coagulative necrosis of hepatocytes. This did not allow for more details of subcellular changes within the liver cells [233]. The sequence of events suggests that PA-HSOS is primarily a vessel disease, followed by injurious manifestations of LSECs and liver cells [231,233]. Time line and mechanistic data of PA-HSOS, however, suggest that at first, the liver cells provide toxic, metabolically formed PAs that subsequently cross the plasma membranes of the hepatocytes and finally bind with proteins of non-liver cells, such as sinusoidal lining cells and LSECs.

### 11.9. Specific Causality Assessment Using RUCAM

RUCAM is worldwide used to assess causality in 95,885 cases of both DILI and HILI [234], with additional details provided for DILI [16,17,18,19] and HILI [42,196,225]. With respect to HILI including PA-HSOS, overall 14,029 RUCAM based cases were published until the mid of 2020 [234], among them were 28 PA-HSOS cases [211,217]. The original RUCAM was published in 1993 [235,236] and is now applicable to DILI and HILI in the updated version published in 2016 [201]. To improve accuracy, RUCAM provides two scales, one for the hepatocellular and one for the cholestatic and/or mixed type of liver injury [201]. Using algorithms that are based on principles of AI (Artificial Intelligence) to solve difficult conditions, such as complex diagnoses in medicine [237], the RUCAM was published as a diagnostic algorithm with the intention to improve and standardise the diagnosis of liver injury cases by avoiding the introduction of errors and subjective opinions, such as those due to global introspection [201,235,236,237]. RUCAM includes AI essentials and is based on seven distinct domains comprizing key elements that are well defined and provide individual sores, shown with the updated RUCAM [201]. The RUCAM scale, specific for the hepatocellular type of liver injury, considers domains of the time to onset from the beginning (or the cessation) of the drug/herb use (scores +2 or +1), course of ALT after cessation of the drug/herb (scores +3 to −2), risk factors (scores +1 or 0), concomitant drug(s) and herb(s) (scores 0 to −3), search for alternative causes (scores +2 to −3), knowledge of product hepatotoxicity (scores +2 to 0) and response to unintentional reexposure (scores +3 to −2) [201]. The broad scoring range reflects the variability of some criteria and allows for the selection of a precise attribution, avoiding a black-or-white choice. With +14 down to −9 points, the final score by products indicates the causality level: score ≤0, excluded causality; 1–2, unlikely; 3–5, possible; 6–8, probable; ≥9, highly probable (Table 6) [201].

As a reminder, many details are available in instructions regarding how best to apply the updated RUCAM and how to handle specific questions and conditions that may emerge during causality assessments using RUCAM [201]. Well-accepted by the scientific community [16,17,234], RUCAM is privileged as a structured, transparent, user friendly, objective, and quantitative diagnostic algorithm specific for liver injury [201]. As with any clinical diagnosis, causes alternative to the suspected PA-HSOS have to be excluded, some of them are listed in the online publication on the updated RUCAM [201]. Alternative diagnoses among an initial PA-HSOS cohort were professionally recognized: Budd Chiari syndrome, DILI, acute or subacute liver failure, decompensated cirrhosis, cardiogenic ascites, and hepatic amyloidosis [202]. Special care is also needed in patients with suspected HSCT-HSOS [6,200]. In general, RUCAM-based causality gradings may be low, especially in patients with PA-HSOS and lacking abnormal serum ALT values, conditions that do not provide scores for all RUCAM elements.

### 11.10. Treatment and Prognosis

Apart from the cessation of PA exposure, symptomatic management of the ascites is the primary therapeutic goal, achieved by restriction of fluid and salt (sodium) intake, a diet rich in proteins, and drug treatment with spironolactone [6]. For refractory ascites, therapeutic options in selected patients may include anticoagulant therapy [6,238,239], transjugular intrahepatic portosystemic shunt (TIPS), and liver transplantation, but positive data derived from RCTs are limited, in part controversially discussed, and require case-by-case decisions due to the lack of an established standard therapy [6]. Independent prognostic factors for patients with PA-HSOS are serum albumin, serum urea, and severity grading, facilitating early intervention in patients at increased risks [240]. Two thirds of the patients with PA-HSOS will recover and one quarter experience a chronic course of the disease (Table 6) [211]. The lethality rate is variable, ranging from 10% to 40%, and due to liver failure [6,211].

## 12. Sporadic PA-HSOS Cases and Small Case Series

Epidemiological evidence is lacking for increased risks of humans consuming regularly low doses of PAs contaminating foodstufff, teas and herbal infusions [241]. However, the consumption of higher amounts of PAs explains the sporadic occurence of PA-HSOS cases, published as single case reports or small case series throughout the world. These cases are likely the tip of an iceberg, because early stages of PA-HSOS are rarely recognized and late stages can easily be misdiagnosed.

Threatening is the occurence of human toxicity by higher amounts of PAs, resulting in embryo or fetal PA-HSOS, described in a preterm newborn with hepatomegaly and fetal ascites, diagnosed upon sonography examination, while the mother underwent a Cesarean section because of risky fetal asphyxia with newborn death shortly after birth [242]. Autopsy described HSOS typical for PA poisoning, confirmed by PA analysis in the liver. A herbal mixture used for cooking in the family showed, upon chemical analysis, high amounts of PAs, suggesting a causal relationship with the disease of the newborn and transfer of PAs from the pregnant woman to the susceptible unborn baby.

A major clinical challenge was a recently published case of a 22 year old female Chinese patient, who was diagnosed with HSOS of unknown etiology, but in retrospect, was likely due to PAs, as she was not on drugs that could have caused HSCT-HSOS [243]. She received a liver transplant, and HSOS recurred twice in the two subsequent liver allografts, caused either by immunesuppressive drugs in connection with post transplantation therapy, or possibly due to an antibody-mediated autoimmune response or genetic susceptibility.

The very early literature on PA-HSOS in adults commonly presents case reports of limited value, often lacking a robust causality assessment, and current biomarker such as PPAs were not available at that time. Considering the experimental studies and PA-HSOS cases published recently [239,244,245,246,247,248,249,250], details of selected cases will be provided. RUCAM was used to assess causality in some of the reports [244,251], but not in others [239,245,246,248,249].

### 12.1. PA-HSOS by Senecio brasiliensis

A 54-year-old female patient with asthenia, malaise, jaundice, ascites and bilateral effusion [244]. Prior to hospital admission, she consumed for 20 days a tea prepared from *Senecio brasiliensis* as prescribed by a local healer for menopausal symptoms. Causality assessment using the updated RUCAM provided a score of six, in line with a probable causality grading. Imaging and liver histology data were suggestive of PA-HSOS. Although the use of *Senecio brasiliensis* was immediately stopped, the clinical course was uneventful due to complications such worsening of the mental status in form of hepatic encephalopathy, acute respiratory distress syndrome requiring endotracheal intubation for mechanical respiration, oliguria demanding hemodialysis, spontaneous bacterial peritonitis, sepsis, mesenteric ischemia, confirmed by explanatory laparotomy and requiring resection of the necrotic small bowel segment. Death occured on day 25 after admission. This case clearly shows the problems of using a PA-containing plant for menopausal symptoms as a minor ailment, for which not only data on RCTs are missing, but also an imbalance exists regarding the benefit–risk constellation.

### 12.2. PA-HSOS Due to Gynura segetum

A case series of five patients with PA-HSOS due to *Gynura segetum* provided a few details on the indications for which ailments or diseases this herbal medicine was used [239]. Indications included vein varicosis of the lower limbs (case 1), joint trauma (case 2), after femoral head replacement (case 3), after spinal trauma (case 4), and waist sprain (case 5). Although all patients met the Nanjing diagnostic criteria, the diagnosis was not verified by RUCAM.

### 12.3. PA-HSOS Related to Gynura segetum

The 68-year-old female patient used *Gynura segetum* for unknown indication but it was mentioned that this TCM plant is commonly used for treating traumatic injury [245]. PA-HSOS was clinically diagnosed, the use of the Nanjing criteria or RUCAM was not mentioned.

### 12.4. PA-HSOS Caused by Gynura segetum

The 46-year-old man had consumed 200 mL spirits on a daily basis for the last 10 years and had recently used large amounts of *Gynura segetum* for traumatic injuries [246]. He was diagnosed with PA-HSOS by *Gynura sgetum*, but the Nanjing criteria or RUCAM were not used to firmly estabilish the diagnosis. It was argued that liver biopsy would have been needed to make a definite diagnosis of HSOS, a proposal not necessarily shared by others. Alcoholic liver disease was not considered as alternative diagnosis [246], although PAs may promote alcoholic liver injury in vitro in normal human liver cells, likely by inducing the inflammatory cytokines and inceasing the apoptotic effects of alcohol [247]. The clinical course was severe and led to the recommendation of a liver transplantation, which was refused by the patient for financial reasons [246].

### 12.5. PA-HSOS by Gynura segetum

The 43-year-old male patient had consumed 450 mL of 30% alcohol, daily, for 20 years and consumed 150 g of medicinal liquor containing Gynura segetum for ten days before developing symptoms [248]. The diagnosis of HSOS combined with alcoholic cirrhosis was made using liver histology. The Nanjing criteria were not applied nor was RUCAM used for assessing causality.

### 12.6. PA-HSOS Attributed to Gynura segetum

The 83-year-old male patient had a long history of using TCMs, such as *Gynura segetum* [249]. The diagnosis of HSOS by this herbal medicine was made from its prolonged use and imaging data. Nanjing criteria and RUCAM were not applied. Liver histology was not available but considered as gold standard for the diagnosis. Reported indications for its use included improving blood circulation, removing blood stasis, and pain relief. It is also used for trauma conditions, bone fractures, and joint diseases. Data of efficacy based on RCTs were not provided.

### 12.7. PA-HSOS Due to Gynura japonica

Substantial progress is evident from recent clinical studies on implementing RUCAM in additional PA-HSOS cases and and evaluations of blood pyrrole-DNA adducts (PDAs) [250]. First, in animals with PA-HSOS caused by *Gynura japonicum*, blood PDAs were found, raising the question whether similar results can be achieved in humans. Second, in a subsequent study comprizing 18 patients with RUCAM based PA-HSOS caused by the consumption of *Gynura japonica* in form of decoction, herbal wine, or ground powder, PDAs were evaluated to diagnose PA genotoxicity and define the early tumorigenic risk in patients [250]. PDAs were found in all patients’ blood samples, collected one month after hospital admission; this is best explained by their reduced adduct degradation. The observation of PDA persistence allows for their use as diagnostic biomarkers of PA genotoxicity.

Other clinical data, including those related more specifically to studies on the updated RUCAM, are highly appreciated and of special interest. For instance, in 11/18 patients (61%) RUCAM scores were 6-8 in line with a probable causality grading, whereas scores of 9–10 were even higher in 7/18 patients (39%), suggesting a highly probable causality grading [250]. The unexpected high RUCAM scores are perfect and reflect a professional study protocol, likely also completeness of data to be included in the RUCAM scale, high LTs, and lack of confounding variables. As another highlight, the liver injury pattern was assessed with one-third each being of the hepatocellular injury, cholestatic injury, and mixed injury type. This RUCAM-based report should be used as an example for other PA-HSOS cases to be evaluated for causality in the future.

Additional demographic details were presented in these patients with PA-HSOS caused by *Gynura japonica* and established diagnosis using the updated RUCAM [250]. Among the 18 patients, there were more males (56%) than females (44%), with clinical manifestation of ascites (100%), abdominal distension (94%), jaundice (62%), hepatomegaly (56%), and right upper quadrant pain (28%). *Gynura japonica* was used for 10–20 days by most of the patients (44%), followed by 28% who used it for 1–2 months. All patients received a treament by anticoagulant medications, 67% of the patients were treated by TIPS. Finally, laboratory tests were given as median values and ranges for ALT (106.95 U/L, range 29.3–1329.3), AST (98.25 U/L, range 22.6–1278.4), total bilirubin (46.30 μmol/L, range 23.3–153.6) and ALP (120.85 U/L, range 49.6–193). The listing of the reference range for each parameter is also perfect. As a result, RUCAM helped provide a solid base for various characteristic features of this disease.

### 12.8. Misdiagnosed PA-HSOS by Petasites hybridus

Concern created ten spontaneous reports presented to the German regulatory agency on assumed PA liver injury by a herbal drug containing *Petasites hybridus* from the Asteraceae familiy, used as an effective prophylaxis of migraine attacks [251,252,253]. However, subsequent re-evalution of the cases revealed overt misdiagnoses [250], confirmed by other studies [251,253]. First of all, the herbal drug under consideration was an extract processed by high supercritical liquid carbon dioxide extraction in astandardized and patented procedure in order to successfully remove PAs, with an ascertained lack of unsaturated PAs in the herbal drug [251,252,253], and this processed herbal medicine was not able producing the typical histological HSOS picture in animals [253]. The missing PAs in the herbal drug clearly rule out any liver disease by unsaturated PAs, a serious point not considered by the physicians or other health care providers at time of submission of their cases [252]. In addition, alternative causes prevailed in the ten cases, RUCAM-based assessment did not support the herbal plant as culprit of the liver injury, and the patients’ liver histology was not compatible with HSOS. Finally, ascites as the cardinal diagnostic feature was not described in any of the nine out of ten assessed cases. *Petasites hybridus* is listed as a PA-containing herbal medicine (Table 3) [56], but in clinical practice, the differentiation of processed products from unprocessed ones is essential.

## 13. Subtropical and Tropical PA-HSOS Outbreaks

For reasons of completeness, a short overview is presented on PA-HSOS outbreaks in tropical and subtropical regions published from 1976 to 2012 [68,127,128,129,130,131,132,133,134,135,254,255,256,257,258,259,260]. Conditions for these cases are different from single case reports or small case series reported in China and other non-tropical regions, where a sophisticated health system is available and prepared to diagnose local PA-HSOS cases using all essential technical tools including imaging methods.

Previous PA-HSOS outbreaks in tropical and substropical countries and regions were commonly late recognized because physicians and other health care providers were rarely familiar with this special type of liver injury, its variability of clinical features, and definite PA sources. Considering countries reporting clusters of PA-HSOS, causes were tentatively ascribed to the consumption of food made from contaminated grain [254,255,256,257,258,259,260] or to drinks and drinking water contaminated with PAs derived from PA-containing plants nearby the quells [130,131,132]. With respect to grain, PAs may contaminate the grain via PA-containing plants or their seeds co-harvested with the grain, or theoretically also through pre-harvest PA uptake by the grain from contaminated soil or water.

For instance, bread may contain PAs, because grain contains seeds of PA-producing PAs co-harvested with the grain. Alternatively, grain itself may have taken up PAs via soil contaminated by PAs derived from nearby PA-producing plants (Figure 4). However, much has been learned and progress has been made, not only in the early recognition of an outbreak, but also in promoting measures to prevent one.

Reports on PA-HSOS correctly classified large scale intoxications by PAs as outbreaks or clusters, and, less correctly, as epidemics or endemics, easily to be diffused with bacterial, viral, or parasitic infections. HSOS cases presenting recent outbreaks were reported from Afghanistan [254,255,259], India [256,257], Tadjistan [258], Ethiopia [68,127,128,129,130,131,132,133,134,135], and Iraq [260].

### 13.1. Afghanistan

In Afghanistan, the first HSOS outbreak was published in 1976 [254], confirmed by another publication in 1978 [259], with a second outbreak reported in 2010 [255]. The first outbreak occurred following a two-year period of severe drought and affected many patients with massive ascites and emaciation [254]. Clinical and pathological studies revealed typical cases of HSOS. The outbreak was caused by bread made from wheat contaminated with PAs from seeds of *Heliotropium* plants. Examination of the 7200 inhabitants from the affected villages showed evidence of liver disease in 22.6% of them. Clinical improvement was observed in thirteen cases after three to nine months, and, in three cases, liver biopsies showed almost complete disappearance of initial abnormalities [254]. Further studies revealed heliotrine as the main PA causing the HSOS [259]. Characteristic morphological findings of the liver showed centrilobular hemorrhagic necrosis, followed by occlusive changes in the hepatic veins, finally resulting in nonportal cirrhosis. The sequence of changes observed suggested primary parenchymal injury and secondary obstructive lesions at the sinusoidal level [259].

### 13.2. India

In India, a minor HSOS outbreak of 25 patients with rapidly developing ascites due to portal hypertension was published [256]. Illness started with mild pain in the epigastrium and the right upper abdominal quadrant, followed by a drop of urinary output and rapidly filling ascites, in all patients, in the absence of jaundice [256]. Liver histology was similar to the report from Afghanistan [259]. In the cohort of India, 11 patients died out of 25 patients retrieved from a population of 350 people [256]. HSOS was probably caused by cereals mixed with seeds of the plant *Crotalaria* spp. containing PAs [257].

### 13.3. Tadjistan

In Tadjistan, the HSOS outbreak included 3906 patients and was ascribed to the use of bread from wheat contaminated with PAs derived from *Heliotropium lasocarpium* and their seeds collected together with the wheat [258]. Clinical features included abdominal pain, nausea or vomiting, and asthenia (stage I), hepatomegaly combined with stage I (stage II), ascites plus features of stages I and II (stage III), and alteration of consciousness (stage IV).

### 13.4. Ethiopia

In Ethiopia, an outbreak of fatal liver disease of unidentified cause led to a case-control epidemiological study collecting information from the affected (case) area and a non-affected adjacent area (control) using a structured questionnaire [261], showing that residents of the affected sites relied more on unprotected or protected wells as a source of drinking water, while most of the non-affected depended on fresh water from a river or unprotected spring. This suggested that the problem of the case area could be linked to the special water source [260]. Subsequent clinical, laboratory, toxicological, and histopathologic studies classified the fatal liver disease as HSOS due to PAs [261] contaminating water from the unprotected well [262], in which the PA-containing plant *Ageratum* sp. abundantly thrived [263]. In addition, PAs were found in Tela, an Ethiopian culture drink similar to beer. Overall, these conditions describe the challenges of finding HSOS and searching for the origin of PAs.

### 13.5. Iraq

In Iraq, a small HSOS outbreak comprizing 14 patients was reported, who consumed wheat accidently contaminated with *Senecio* seeds containing PAs [264]. Clinical presentation of this cohort included ascites (100%), abdominal pain (100%), hepatomegaly (57%), vomiting (50%), jaundice (43%), and splenomegaly (36%). LTs were slightly increased: bilirubin 4.6 mg (normal 0.2–1.0), ALT 51.9 U/L (5–20), and AST 37.4 U/L (5–20). Children were at higher risk. Among the 14 patients, the overall fatality rate was 14.3%, resulting from liver failure and bleeding esophageal varices [264].

### 13.6. Uganda

In Uganda, liver fibrosis as assessed with transient elastography (FibroScan^®^) was found in 19 patients with and without human immunodeficiency virus (HIV) infections, who used a variety of PA-containing plants preferentially from the Asteraceae, Fabaceae, and Lamiaceae families [265]. The shortcoming of this study is its failure to use RUCAM, the criteria of PA-HSOS were not available at that time, and PPAs were not known. While liver fibrosis can easily and non-invasively be assessed, there are no studies how liver fibrosis correlates with PA-HSOS with fibrosis as one of the typical liver histology features in humans [211] reproducible in animal models [204,232].

## 14. Photography of PA-Containing Plants

**Figure 10:**
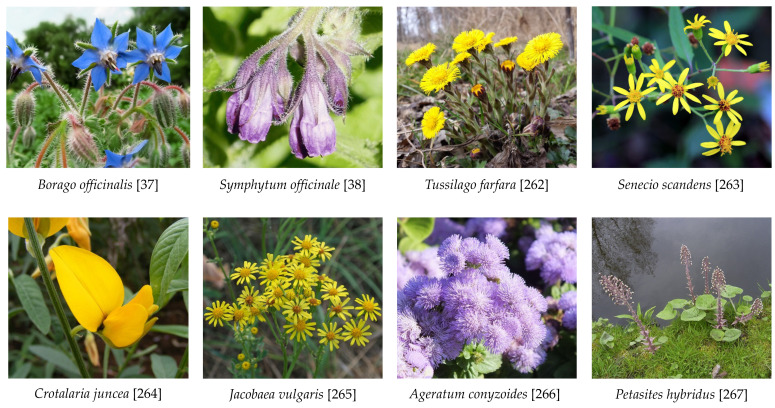
Photography of PA-containing plants. All pictures above show PA-containing plants mentioned in the text of this manuscript and are obtained from open access publications as referenced [37,38,262,263,264,265,266,267]. *Symphytum officinale* (syn. *Symphytum peregrinum*), *Petasites hybridus* (syn. *Petasites officinalis*), *Tussilago farfara* (syn. *Tussilago alpestris*), and *Jacobaea vulgaris* (syn. *Senecio jacbaea*) are native to Europe. *Senecio scandens* (syn. *Senecio chinensis*) has a native range from China to Indo–China and Central Malesia, meanwhile that of *Crotalaria juncea* (syn. *Crotalaria fenestrate*) is from Afghanistan to Indo-China. *Ageratum conyzoides* (syn. *Ageratum album*) mostly distributes in Tropical America, whereas *Borago officinalis* (syn. *Borago hortensis*) originates from the Mediterranean region.

Plants containing PAs are distributed all over the world, and many of these present fascinating flowers with typical appearance and color. As a result, they can easily be identified in the free landscape as well as in cornfields or on meadows where cattle is grazing or bees are flying around. Early recognition of plants containing PAs will help remove them before they are incorporated in products for human use or as feed for cattle. Out of the many plants discussed in this article, photographies of a few plants are presented *as examples: Borago officinalis, Symphytum officinalis, Tussilago farfara, Senecio scandens, Crotalaria juncea, Jacobaea vulgaris, Ageratum conyzoides,* and *Petasites hybridus* (Figure 10). 

## 15. Conclusions

PAs are synthetisized by homospermidine synthase in plants, used as herbal medicines, or detected as contaminants in foodstuffs and drinking water. While saturated PAs are commonly harmless, 1,2-unsaturated PAs may become toxic if converted to toxifying PA radicals by removing the double bond between C1 and C2, affecting also the liver and causing PA-HSOS. The conversion to PA radicals occurs in the endoplasmic reticulum, corresponding to the microsomal fraction of the hepatocytes. The toxifying reaction is dependent on NADPH and microsomal CYP with its isoforms CYP 2A6, CYP 2A4, and CYP 3A5; it also requires molecular oxygen, generating toxic radicals in line with ROS due to incomplete oxygen split. PA radicals bind to hepatocellular or blood proteins and produce pyrrolizidine protein adducts, detectable in the blood, clinically appreciated as diagnostic biomarkers in support of the updated RUCAM, assessing causality for PAs and the diagnostic Nanjing criteria. Part of the PA radicals enter and injure the LSECs, lacking, themselves, CYP isoforms for their own metabolic toxification of 1,2-unsaturated PAs. PA-HSOS is dependent on high, daily and cummulative doses, allowing for animal models with results that can be well-translated to human conditions.

PA-HSOS is primarily a vessel disease of the liver. The diameters of the veins within or approaching the liver are decreased, reducing the blood flow into the liver and causing a backlog by extravasation of fluids into the abdominal cavity, clinically known as ascites, the leading diagnostic feature. LTs like ALT or AST are variable, either within the normal range or moderately increased, conditions that impede early diagnosis of incipient PA-HSOS. It does not seem that the users of small amounts of unsaturated PAs found in foodstuffs in Western countries are at risk of PA-HSOS. On the contrary, following the consumption of larger amounts of PAs cases of PA-HSOS were published as single case reports, small series, or case clusters. Clusters of PA-HSOS were due to foodstuff and drinking water contaminated with unsaturated amounts of PAs, published from countries like Afghanistan, India, Tadjistan, Ethiopia, and Iraq. Single case reports came from several countries, case series preferentially from China, where intoxications by the PA-containing *Gynura segetum* prevailed. Intoxicant causes were preferentially retrieved from plants from the following families: Ariaceae (*Castilleja* spp.), Boraginaceae (*Heliotropium* spp. *Trichodesma* spp., *Symphytum* spp. such as Comfrey), Compositae (*Senecio* spp. like Bush tea, *Eupatorium* spp.), and Leguminosae (*Crotalaria* spp.). Further studies on its relevance under field conditions are required, for the fascinating Selmar concept of horizontal PA transfer from PA-producing PAs reaching the soil and being taken up by non-PA-containing plants via their roots.

Apart from cessation of PA exposure, symptomatic management of the ascites is the primary therapeutic goal. For refractory ascites, therapeutic options may include anticoagulant therapy, transjugular intrahepatic portosystemic shunt, and liver transplantation, but robust data derived from RCTs are limited. Independent prognostic factors for patients with PA-HSOS are serum albumin, serum urea, and severity grading, facilitating early intervention in patients at risks. Two thirds of the patients with PA-HSOS will recover and one quarter will experience a chronic course of the disease. The lethality rate, ranges from 10% to 40% and is due to liver failure. For the future, prevention of PA-HSOS should be the main goal to reduce the number of PA-HSOS patients, and correct diagnosis using the updated RUCAM for causality assessment is essential.

In essence, plants produce, via the homospermidine synthase, various unsaturated PAs, representing multicyclic chemicals with a double bond between C1 and C2, which is broken up in the human liver via the dehydrogenation pathway into toxic radical DHPAs, culprits for the initiation and perpetuation of the severe liver injury called HSOS.

## Figures and Tables

**Table 1:** Amounts of PAs in feed products.

Feed Products	Mean PA Amounts (µg/kg)		
	Lower Bound	Middle Bound	Upper Bound
Wheat	23	171	320
Maize	0	151	302
Millet	0	151	302
Oats	0	151	302
Rice, broken	0	151	302
Sorghum, Milo	0	151	302
Palm kemel expeller	0	151	302
Rape seed	9	159	308
Toasted soya beans	3	153	303
Sunflower seeds	5	155	305
Linseed	30	177	325
Peas	16	166	315
Carob, dried	8	156	305
Sweet lupines	0	151	302
Carrots	0	151	302
Citrus pulp	12	161	311
Lucerne (alfalfa)	368	503	637
Grass, field dried, hay	174	322	470

Mean values of the PAs reported for different types of feed samples collected in the Netherlands. PAs are not necessarily synthesized by the plant itself. Data were given as total amounts of PAs, combining saturated and 1,2-unsaturated PAs. The table is derived from the EFSA (European Food Safety Authority), 2017 [23]. Abbreviation: PA, pyrrolizidine alkaloid.

**Table 2:** Amounts of PAs in plant products for human use.

Plant Products for Human Use	Mean PA Amounts (µg/kg)		
	Lower Bound	Middle Bound	Upper Bound
Herbal mix	353	492	630
Artichoke	2252	2385	2517
Camomile	35	184	334
Dandelion	663	793	924
Fennel	1592	1732	1871
Ginseng	5	154	302
Goldenrod	18	165	312
Knotweed	97	241	385
Leek	0	151	302
Mangold	0	151	302
Milk thistle	12	161	309
Mint	0	151	302
Nettle	16	165	314
Oregano	89	235	381
Parsley	0	151	302
Rose tip	0	151	302
Rosemary	5	154	302
Verbena	18	164	310

Amounts of PAs, combining saturated and 1,2-unsaturated PAs, not necessarily synthesized by the plant itself. The data were retrieved from EFSA, 2017 [23]. Abbreviation: PA, pyrrolizidine alkaloid.

**Table 3:** Examples of medicinal plants containing variable PA compounds.

Plant (Family)	Compounds of Pyrrolizidine Alkaloids (Selected)
*Alkanna* (Boraginaceae)	7-Angeloylretronecine [57,58], 9-Angeloylretronecine [57], 7-Tigloylretronecine [57], 7-Senecioylretronecine [57], 7-Tigloylretronecine [57], 9-Senecioylretronecine [57], 7-Angeloyol-9-(hydroxypropenoyl) retronecine [57], 7-Tigloyl-9-(hydroxy propenoyl) retronecine [57], 7-Angeloyol-9-(2,3-dihydroxypropanoyl) retronecine [57], 7-Tigloyl-9-(2,3-dihydroxypropanoyl) retronecine [57], Triangularine [57,58], Triangularicine [57], Dihydroxytrian gularicine [57,58]
*Anchusa* (Boraginaceae)	Anthamidin [57], Supinine [57,59], Intermedin [57,59], Lycopsamine [57,59], Currassavine [59]
*Borago* (Boraginaceae)	Lycopsamine [60], Supinidine [60], Viridiflorate [60], Cynaustine [60], Amabaline [60,61], Thesinine [61,62]
*Brachyglottis* (Asteraceae)	Senecionine [63,64], Retrorsine [63,64], Integerrimine, Senkirkine [63], Hectorine [63,64], Petasinine [63,64]
*Cineraria* (Asteraceae)	Otosenine [65,66], Florosenine [65,66], Floridanine [65,66], Doronine [65], Senecionine [66,67], Integerrimine [66,67], Seneciphylline [66,67]
*Crotalaria* (Leguminosae)	Tashiromine [68], Retronecine [68], Heliotridine [68], 9-Isosenecioylretronecine [68], 9-Angeloylretronecine [68], 7-Seneciolylretronecine [68], 9-Hydroxyheptanoylretronecine [68], 9-Hydroxyisohexenoylretronecine [68], Rinderine [68], 9-Hydroxytigloylretronecine [68], Monocrotaline [68], Dihydrosenecionine isomer [68]
*Cynoglossum* (Boraginaceae)	Heliosupine [69], Rinderine [69], Echinatine [69], Viridiflorine [69]
*Eupatorium* (Asteraceae)	Lindelofine [70], Supinine [70,71,72,73], Lycopsamine [73,74,75], Intermedin [73,74,75], Amabaline [71,76], Echinatine [71,76], Rinderine [71], Viridiflorine [71], Cynaustraline [71], Tussilagine [76]
*Foeniculum vulgare* (Apiaceae)	PA compounds not individually specified [77]
*Heliotropium* (Boraginaceae)	Trachelanthamine [78], Floridine [78], Heliovicine [78], Lycopsamine [79,80], Amabiline [79], Curassavine [79,81], Heliospathine [79], Intermedin [80], Europine [82,83,84,85], Liamin [82], Heliotrine [84,85,86], Lasiocarpine [84], Retronecine [80,87,88,89], Helibracteatine [87], Helifoline [88], Heliscabine [89], Heliosupine [86], Echinatine [86], Supinine [85], Heleurine [85], Coromandaline [81]
*Lithospermum* (Boraginaceae)	Lithosenine [90], Lycopsamine [91], Canescine derivatives [91,92], Canescinine [91], Intermedine [91,93], Mysocorpine [93]
*Petasites* (Asteraceae)	Senkirkine [94,95], Senecionine [94,96], Integerrimine [96], Petasitenine [95], Neopetasitenine [95]
*Senecio* (Asteraceae)	Ridelline [97,98,99,100,101,102,103], Retrorsine [97,98,99,100,101,102,103], Seneciphylline [97,98,99,100,101,102,103], Senecionine [97,98,99,100,101,102,103], Senkirkine [97,98,99,102], Jacobine [104,105], Integerrimine [98,99,100,101,103], Spartiodine [98,99,100,103], Senecivernine [99,100,103,104,105], Platyphylline [97,98], Usaramine [97,99,103,104,105], Adinofoline [98,104], Florosenine [104], Erucifoline [104], Otosenine [104], Triangularine [106,107], 7-Angeloylheliotridine [108,109], Uspallatine [110], Rosmarinine [111,112], Angularine [111,112], Hadiensiene [111], Ruwenine [113], Ruzorine [113], Doriasenine [114], Sceleratine [115]
*Symphytum* (Boraginaceae)	Echimidine [116,117,118], Symphytine [116,117], Lasiocarpine [101], Intermedin [119], Lycopsamine [118,119]
*Tussilago* (Asteraceae)	Senkirkine [120,121], Senecionine [121], Intergerrimine [121], Seneciphylline [121], Senecivernine [122]

The listed PAs include saturated PAs and 1,2-unsaturated PAs. Most of the data and references were obtained from the open access report of Kopp et al. [56], which provided additional references and details of interest.

**Table 4:** Most active human liver CYP isoforms metabolizing unsaturated PAs.

PA Type	Most Active CYP Isoforms
Clivorine	CYP 3A4
Integerrimine	CYP 3A4, CYP 3A5
Lasiocarine	CYP 3A4, CYP 3A5
Monocrotaline	CYP 2A6
Retrorsine	CYP 2A6, CYP 3A4, CYP 3A5
Riddelline	CYP 3A4, CYP 3A5
Senecionine	CYP 2A6, CYP 3A4, CYP 3A5
Seneciphylline	CYP 2A6, CYP 3A4, CYP 3A5
Senkirkine	CYP 3A4

The most active CYP isoforms catalyze the formation of pyrrole protein adducts. Abbreviations: CYP, cytochrome P450; PA, pyrrolizidine alkaloid. Updated, adapted, and modified from a previous report by He et al. [172].

**Table 5:** Clinical characteristics of the HSOS caused by *Gynura segetum* containing unsaturated PAs.

Parameter	Results
Cohort	*n* = 116
Gender	Males 57 (50.4%) Females 56 (49.6%) (NA 3)
Age	17–76 years
Ascites	115/116 cases (99.1%)
Hepatomegaly	104/113 cases (92.0%)
Jaundice	95/113 cases (84%)
ALT elevation	47/60 cases (78.3%) (NA 56 cases)
AST elevation	50/58 cases (86.2%) (NA 58 cases)
Outcome	Recovery 75 cases (66.4%) Chronicity 27 cases (23.9%) Death 11 cases (9.7%) (NA 3 cases)

Data represent typical features of PA-HSOS cases, with ascites as leading diagnostic symptom. Cases were not assessed regarding causality for unsaturated PAs. Details were collected from published reports with individual references [211]. Abbreviations: ALT, alanine aminotransferase; AST, aspartate aminotransferase; hepatic sinusoidal obstructive syndrome NA, not available; PAs, pyrrolizidine alkaloids. Adapted and modified from a previous open access report [202] and modified from data published by Gao et al., 2012 [211].

**Table 6:** The updated RUCAM scale for the hepatocellular injury.

Suspected Product	Date	
Items for Hepatocellular Injury	Score	Result
**1. Time to onset from the beginning of the drug/herb consumption** •5–90 days (rechallenge: 1–15 days) •<5 or >90 days (rechallenge: >15 days) Alternative: Time to onset from cessation of the drug/herb •≤15 days (except for slowly metabolized chemicals: >15 days)	+2 +1 +1	□ □ □
**2. Course of ALT after cessation of the drug/herb** **Percentage difference between ALT peak and ULN** •Decrease ≥50% within 8 days •Decrease ≥50% within 30 days •No information or continued drug use •Decrease ≥50% after the 30^th^ day •Decrease <50% after the 30^th^ day or recurrent increase	+3 +2 0 0 −2	□ □ □ □ □
**3. Risk factors** •Alcohol use (current drinks/d: >2 for women, >3 for men) •Alcohol use (current drinks/d: ≤2 for women, ≤3 for men) •Age ≥55 years •Age <55 years	+1 0 +1 0	□ □ □ □
**4. Concomitant drug(s)/herb(s)** •None or no information •Concomitant drug/herb with incompatible time to onset •Concomitant drug/herb with time to onset 5–90 days •Concomitant drug/herb known as hepatotoxin and with time to onset •5–90 days •Concomitant drug/herb with evidence for its role in this case (positive rechallenge or validated test)	0 0 −1 −2 −3	□ □ □ □ □
**5. Search for alternative causes** **Group I (7 causes)** •HAV: Anti-HAV-IgM •HBV: HBsAg, anti-HBc-IgM, HBV-DNA •HCV: Anti-HCV, HCV-RNA •HEV: Anti-HEV-IgM, anti-HEV-IgG, HEV-RNA •Hepatobiliary sonography/Doppler/CT/MRC •Alcoholism (AST/ALT ≥2) •Acute recent hypotension history (particularly if underlying heart disease) **Group II (5 causes)** •Complications of underlying disease(s), such as sepsis, metastatic malig-nancy, autoimmune hepatitis, chronic hepatitis B or C, primary biliary cholangitis or sclerosing cholangitis, genetic liver diseases •Infection suggested by PCR and titer change for CMV (anti-CMV-IgM, anti-CMV-IgG) •EBV (anti-EBV-IgM, anti-EBV-IgG) •HSV (anti-HSV-IgM, anti-HSV-IgG) •VZV (anti-VZV-IgM, anti-VZV-IgG) **Evaluation of groups I and II** •All causes-groups I and II—reasonably ruled out •The 7 causes of group I ruled out •6 or 5 causes of group I ruled out •Less than 5 causes of group I ruled out •Alternative cause highly probable	Tick if negative □ □ □ □ □ □ □ □ □ □ □ □ +2 +1 0 −2 −3	Tick if not done □ □ □ □ □ □ □ □ □ □ □ □ □ □ □ □ □
**6. Previous hepatotoxicity of the drug/herb** •Reaction labelled in the product characteristics •Reaction published but unlabelled •Reaction unknown	+2 +1 0	□ □ □
**7. Response to unintentional reexposure** •Doubling of ALT with the drug/herb alone, provided ALT below 5 × ULN before reexposure •Doubling of ALT with the drug(s)/herb(s) already given at the time of first reaction •Increase of ALT but less than ULN in the same conditions as for the first administration •Other situations	+3 +1 −2 0	□ □ □ □
**Total score of the patient under consideration through combining individual scores:**		

The above items specifically refer to the hepatocellular injury rather than to the cholestatic and/or mixed liver injury. Squared boxes above require either inclusion of a score related to the patient or a ticking if negative or not done. Abbreviations: ALT, alanine aminotransferase; AST, aspartate aminotransferase; CMV, cytomegalovirus; CT, computer tomography; EBV, Epstein Barr virus; HAV, hepatitis A virus; HBc, hepatitis B core; HBsAg, hepatitis B antigen; HBV, hepatitis B virus; HCV, hepatitis C virus; HEV, hepatitis E virus; HSV, herpes simplex virus; MRC, magnetic resonance cholangiography; RUCAM, Roussel Uclaf Causality Assessment Method; ULN, upper limit of the normal range; VZV, varicella zoster virus. Total score and resulting causality grading: ≤0, excluded; 1–2, unlikely; 3–5, possible; 6–8, probable; ≥9, highly probable. The table was retrieved from an earlier open access publication by Danan and Teschke [201].

## Data Availability

All data presented and discussed in this article were derived from and are available in the referenced reports.
